# Timing and Pattern of Early Diversification in Drosophilidae (Diptera)

**DOI:** 10.1093/molbev/msaf269

**Published:** 2025-10-23

**Authors:** Guilherme Rezende Dias, Eduardo Guimarães Dupim, Thyago Vanderlinde, Beatriz Mello, Antonio Bernardo Carvalho

**Affiliations:** Departamento de Genética, Universidade Federal do Rio de Janeiro, Rio de Janeiro 21941-617, Brazil; Departamento de Genética, Universidade Federal do Rio de Janeiro, Rio de Janeiro 21941-617, Brazil; Departamento de Genética e Biologia Evolutiva, Instituto de Biociências, Universidade de São Paulo, SP 05508-090, São Paulo, Brazil; Departamento de Genética, Universidade Federal do Rio de Janeiro, Rio de Janeiro 21941-617, Brazil; Departamento de Genética, Universidade Federal do Rio de Janeiro, Rio de Janeiro 21941-617, Brazil; Departamento de Genética, Universidade Federal do Rio de Janeiro, Rio de Janeiro 21941-617, Brazil

## Abstract

Despite their relevance as model organisms, the early diversification patterns in Drosophilidae remain poorly resolved, with most studies focusing on *Drosophila*. Here, we employed a phylogenomic framework for 33 taxa: 27 drosophilid species representing most tribes of both subfamilies (Drosophilinae and Steganinae) plus 6 taxa from other families of Ephydroidea (Braulidae, Cryptochetidae, Curtonotidae, and Ephydridae). Besides inferring phylogenetic relationships, we estimated divergence times and substitution rates using a fossil-calibrated Bayesian approach. Our results recover Drosophilinae as monophyletic (among the taxa sampled) but place *Braula coeca* (Braulidae) within Steganinae, rendering Drosophilidae nonmonophyletic and underscoring the need for taxonomic revision. Relationships within Steganinae (including *Braula*) were fully resolved, whereas the position of some Drosophilinae lineages (eg *Scaptodrosophila*) remains uncertain, likely due to extensive gene tree heterogeneity. Divergence time estimates suggest that the family originated near the Cretaceous–Paleogene boundary (67.3 Ma; 95% highest posterior density: 83 to 52 Ma), with subfamilies diversifying primarily during the Eocene (56 to 34 Ma). The neutral evolutionary rate, estimated from fossil calibrations and third codon positions, aligns with previous biogeographically calibrated estimates but is lower than mutation-derived rates, likely reflecting the action of purifying selection and uncertainty about generation times across lineages.

## Introduction

Drosophilids are a central model system in evolutionary biology, with studies across diverse fields relying on phylogenetic hypotheses for this group (eg Castillo 2017; Prieto-godino et al. 2017; Rice et al. 2018). However, most research on Drosophilidae phylogenetics has focused on the genus *Drosophila*, leaving the relationships among other lineages—especially within Steganinae—largely unresolved (O’Grady and DeSalle 2018).

Traditionally, Drosophilidae has been divided into 2 subfamilies: Drosophilinae (∼3,300 species) and Steganinae (∼700 species) (Brake and Bächli 2008). This division, based on morphological features (Throckmorton 1975; Okada 1989; Grimaldi 1990), has been inconsistently supported by molecular data (Remsen and O’Grady 2002; van der Linde et al. 2010; Yassin 2013; Dias et al. 2020; Finet et al. 2021; Suvorov et al. 2022; Kim et al. 2024). In addition, both subfamilies have been subdivided into tribes, but their monophyly and composition remain highly contentious (Okada 1989; Grimaldi 1990; van der Linde et al. 2010; Russo et al. 2013; Yassin 2013). As a result, the evolutionary origins and early diversification within Drosophilidae remain poorly understood.

Genomic-scale data offer a powerful tool to resolve these longstanding uncertainties (eg Misof et al. 2014; Song et al. 2012; Stiller et al. 2024; Zuntini et al. 2024), and recent phylogenomic studies have provided evidence supporting the monophyly of both Drosophilinae and Steganinae (Dias et al. 2020; Suvorov et al. 2022; Bastide et al. 2024; Kim et al. 2024). Among these, Kim et al. (2024) dramatically expanded genomic coverage by assembling over 360 drosophilid genomes, including many previously unsampled lineages. However, their study focused on generating genome resources and broad-scale topology, mainly within the genus *Drosophila*, without explicitly addressing early diversification patterns or relationships with other Ephydroidea families.

Another underexplored aspect of drosophilid evolution is the timing of divergence events. Most molecular clock studies have focused on *Drosophila* and relied on biogeographic calibrations based on the formation of the Hawaiian Islands (Russo et al. 1995, 2013; Tamura et al. 2004; Gao et al. 2011; Obbard et al. 2012; Katoh et al. 2017; Suvorov et al. 2022). This strategy has been shown to produce highly uncertain and potentially inflated divergence time estimates (Obbard et al. 2012), and consequently the temporal framework for the diversification of other Drosophilidae lineages remains largely unresolved.

Here, we focus explicitly on the early diversification of Drosophilidae, with particular attention to underrepresented and taxonomically unstable genera from both subfamilies. To this end, we generated new genomic data for 14 species and assembled a phylogenomic dataset comprising 33 species—including representatives from 12 of the 15 tribes proposed to date (Okada 1989; Grimaldi 1990; Yassin 2013). Our sampling strategy prioritizes phylogenetic breadth outside the *Drosophila* genus to better resolve relationships among early-diverging lineages. We use these data to infer both phylogenetic relationships and divergence times, incorporating fossil calibrations, and provide a comparative analysis with previous estimates. Additionally, we include representatives from other Ephydroidea families to reassess their relationships with Drosophilidae.

Our results support the monophyly of Drosophilinae but not of Drosophilidae, due to the placement of *Braula coeca* (Braulidae) within Steganinae. We estimate that the common ancestor of drosophilids likely originated near the Cretaceous–Paleogene (K–Pg) boundary and that most of the diversification of extant genera occurred during the Eocene. While our data support fully resolved Steganinae relationships, some lineages within Drosophilinae remain unstable, particularly *Scaptodrosophila*. Cryptochetidae is recovered as the sister group to Drosophilidae, and our results do not support the monophyly of most currently proposed tribes in either subfamily, underscoring the need for a broad systematic reevaluation of Drosophilidae taxonomy.

## Results and Discussion

**Fig. 1.**
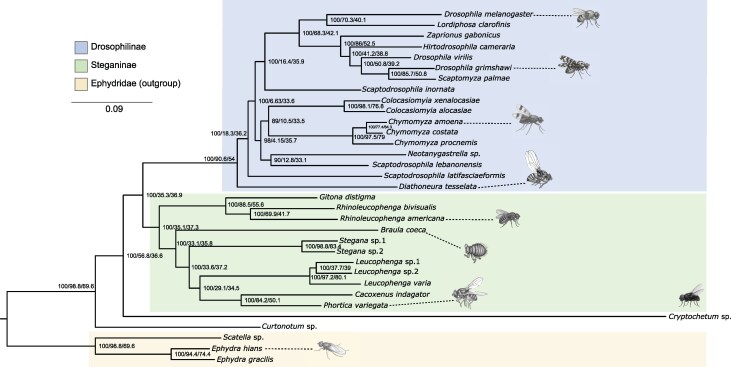
Drosophilidae species tree inferred from the “comprehensive” dataset (1,606 genes, 2,453,970 sites) by concatenation approach using only the first and second codon positions (analysis 2 in Table 1). The outgroup and the subfamilies Steganinae and Drosophilinae are highlighted in orange, green, and blue, respectively. Numbers close to the nodes represent bootstrap/gCF/sCF. Branch lengths are in units of substitutions per site. Representative fly images were generated from reference photographs using a generative AI tool (ChatGPT and OpenAI) and are intended solely for illustration.

Our phylogenomic analyses investigated the relationships among 27 Drosophilidae species (10 Steganinae and 17 Drosophilinae) and 6 species belonging to 4 other Ephydroidea families—namely, 1 from Braulidae, 1 from Cryptochetidae, 1 from Curtonotidae, and 3 from Ephydridae. Ephydridae species were used as outgroups (Wiegmann et al. 2011; Bayless et al. 2021; Winkler et al. 2022). Our main analyses were based on a comprehensive dataset including 1,606 orthologs from 33 taxa, but additional analyses using more restrictive gene inclusion thresholds (ie “conservative” and “relaxed” datasets, see Material and Methods) supported the topology inferred from the comprehensive dataset. All the phylogenetic analyses and results are presented in Table 1, Fig. 1, and Figs. S1 to S18.

**Table 1.** Phylogenomic analyses performed using distinct datasets

Analysis	Data type	Partitioning scheme	Substitution model selection	Species tree inference	Alignment length
Comprehensive (1,606 genes)					
1	Nucleotides	By gene	Per gene	Concatenation: IQTree	3,680,955
2	Nucleotides (1 + 2)	By gene	Per gene	Concatenation: IQTree	2,453,970
3	Nucleotides	n/a	n/a	MSC: ASTRAL-III	n/a
4	Nucleotides (1 + 2)	n/a	n/a	MSC: ASTRAL-III	n/a
Relaxed (1,252 genes)					
5	Nucleotides	By gene	Per gene	Concatenation: IQTree	1,480,434
6	Nucleotides	By codon position (1 + 2 and 3)	Per gene	Concatenation: IQTree	1480,434
7	Nucleotides (1 + 2)	By gene	Per gene	Concatenation: IQTree	989,956
8	Nucleotides	n/a	n/a	MSC: ASTRAL-III	n/a
9	Nucleotides (1 + 2)	n/a	n/a	MSC: ASTRAL-III	n/a
10	Nucleotides	By gene	Per gene + GHOST	MSC: ASTRAL-III	n/a
11	Amino acids	n/a	n/a	MSC: ASTRAL-III	n/a
Conservative (448 genes)					
12	Nucleotides	By gene	Per gene	Concatenation: IQTree	529,665
13	Nucleotides	By codon position (1 + 2 and 3)	Per gene	Concatenation: IQTree	529,665
14	Nucleotides (1 + 2)	By gene	Per gene	Concatenation: IQTree	353,110
15	Nucleotides	n/a	n/a	MSC: ASTRAL-III	n/a
16	Nucleotides (1 + 2)	n/a	n/a	MSC: ASTRAL-III	n/a
17	Nucleotides	By gene	Per gene + GHOST	MSC: ASTRAL-III	n/a
18	Amino acids	n/a	n/a	MSC: ASTRAL-III	n/a

**Table 2.** Comparisons of the divergence dates (Ma) estimated for drosophilids

Study	*Drosophila virilis*—*Scaptomyza*^a^	*Sophophora–Drosophila*	Drosophilidae MRCA	Calibration scheme
This study	21.4 (26.6 to 16.6)	36.5 (45.2 to 28.2)	67.3 (83.5 to 51.8)	Fossil-based
Suvorov et al. (2022) ^b^	30.1 (32.7 to 27.7)	46.8 (49.8 to 43.8)	63.2 (65.7 to 59.8)	Fossil-based
Katoh et al. (2017)	36 (42.8 to 34.2)	NA	NA	Secondary calibrations + biogeography
Russo et al (2013)	37.6 (43.5 to 32.3)	56.4 (66.3 to 48.6)	70.4 (85.9 to 58.9)	Fossils + biogeography
Obbard et al. (2012) ^c^	13 (17 to 9.6)	32 (40 to 25)	NA	Substitution rate (4-fold sites)
Obbard et al. (2012) ^d^	12 (15 to 8.8)	26 (32 to 21)	NA	Biogeography (Hawaiian shield formation)
Obbard et al. (2012) ^e^	47 (79 to 21)	103 (170 to 47)	NA	Biogeography (Hawaiian volcano emergence)
Gao et al. (2011)	49.5 (49.9 to 49.1)	62.9^f^	NA	Secondary calibration from Tamura et al. (2004)
Tamura et al. (2004)	42.9 (51.6 to 34.2)	62.9 (75.3 to 50.5)	NA	Biogeography
Russo et al. (1995)	33.1 (36.3 to 29.9)	39.2 (42.55 to 35.85)	NA	Biogeography

NA, not available.

^a^In Obbard et al. (2012), Russo et al. (1995), and Tamura et al. (2004), we used the age of the MRCA of *virilis*/*repleta*–Hawaiian drosophilids as the same of the *Drosophila virilis–Scaptomyza*. We considered *Scaptomyza* to be in the same clade as the Hawaiian Drosophila based on O'Grady and DeSalle (2018), Russo et al. (2013), and Yassin (2013).

^b^Results obtained from calibration scheme A.

^c^Using only 4-fold degenerate codons. The prior of the rate of the third position was constrained to the estimate provided by Keightley et al. (2009).

^d^Using all codons and a Hawaiian calibration that associates speciation dates with the estimated completion of the first shield for that island.

^e^Using all codons and a Hawaiian calibration that associates speciation dates with the estimated emergence of the first volcano above the surface.

^f^Calibration adopted from Tamura et al. (2004).

We found that the GC content (ie the proportion of guanine and cytosine bases in the sequence) at the third codon position (GC3) is highly heterogeneous across species, whereas the first + second codon positions (GC12) are more homogeneous (Table S1; Figs. S19 and S20). The GC heterogeneity is statistically significant for both GC3 and GC12 (GC3: *F*_32,44975_ = 1,809.3, *P* < 10^−6^ ; GC12: *F*_32,44975_ = 105.7, *P* < 10^−6^ ; one-way analysis of variance [ANOVA]). However, GC3 is much more heterogeneous across species than GC12: the effect size of the species (ie the amount of GC variance explained by species differences, as measured by the squared multiple *R*) is much higher for GC3 (56%) than for GC12 (7%). While these findings are somewhat expected (substitutions in the third codon positions are frequently neutral or quasi-neutral and hence are more susceptible to mutation bias), they are relevant because most phylogenetic methods assume “stationary base composition,” ie an approximately homogeneous GC composition.

Given this strong heterogeneity in GC content of the third codon positions, we focused the discussion on the results using only the first and second codon positions. Besides 1,000 ultrafast bootstrap replicates, we also computed gene concordance factors (gCFs), based on maximum likelihood (ML) gene trees, and site concordance factors (sCFs) for each branch (Minh et al. 2020).

### Phylogenetic Relationships of Drosophilids

Our phylogenomic analyses recovered Drosophilidae as a nonmonophyletic clade, with *B. coeca* (traditionally placed in Braulidae) nested within Steganinae. This corroborates previous studies that have grouped *Braula* within Drosophilidae (Bayless et al. 2021; Winkler et al. 2022; Bastide et al. 2024), though limited sampling of Steganinae previously hindered finer resolution. In our analysis, consistent with Bastide et al. (2024), *Braula* clustered with *Stegana*, *Leucophenga*, *Phortica*, and *Cacoxenus*.

The relationships between Drosophilidae and other Ephydroidea families were consistent with previous molecular studies (Wiegmann et al. 2011; Bayless et al. 2021; Winkler et al. 2022). We recovered *Cryptochetus* (Cryptochetidae) as sister to Drosophilidae, and *Curtonotum* (Curtonotidae) as sister to the clade uniting Drosophilidae and Cryptochetidae.

The classical division of Drosophilidae into Steganinae and Drosophilinae was supported in all analyses. Although this division has occasionally been challenged by studies with limited genetic data (eg van der Linde et al. 2010; Russo et al. 2013), our extensive dataset reinforces it. These previous discrepancies may be explained by the presence of extensive gene tree heterogeneity. For instance, only ∼35% of the 1,606 gene trees of the “comprehensive” dataset recovered Steganinae + Braulidae as a monophyletic group (Fig. 1), likely due to incomplete lineage sorting (ILS) and introgression (Dias et al. 2020).

Within Steganinae, we recovered two major clades: one containing *Rhinoleucophenga* and *Gitona*, and another grouping *Braula*, *Stegana*, *Leucophenga*, *Phortica*, and *Cacoxenus*. This structure is consistent with Bastide et al. (2024) and Kim et al. (2024) and partially supports Throckmorton's (1975) early proposition of a *Stegana–Leucophenga–Phortica* lineage. Notably, we did not recover the monophyly of *Stegana* + *Leucophenga*, instead finding *Leucophenga* more closely related to *Phortica* and *Cacoxenus*. The close relationship between *Rhinoleucophenga* and *Gitona*, first proposed based on morphological traits (Brake and Bächli 2008), was also recovered here.

Drosophilinae has been more extensively studied than Steganinae, but the relationships among its genera have also been controversial in both morphology and molecular-based studies (Okada 1989; Grimaldi 1990; DeSalle and Grimaldi 1991; Remsen and O’Grady 2002; Da Lage et al. 2007; van der Linde et al. 2010; Russo et al. 2013; Yassin 2013; Finet et al. 2021; Suvorov et al. 2022; Kim et al. 2024). This suggests that phylogenetic inference within this subfamily may have intrinsic difficulties, due to biological phenomena such as convergence and retention of polymorphisms (for morphological studies) and extensive ILS and introgression (for molecular studies). We were able to cope with some of these issues using a phylogenomic approach, but our efforts were insufficient to fully resolve the phylogeny of Drosophilinae since the position of some lineages remained uncertain.

Our results suggest *Diathoneura* lineage emerged early in the diversification of Drosophilinae, contrasting with Yassin's (2013) proposal to reclassify it within Ephydridae. We support its retention in Drosophilidae, as previously argued by Grimaldi (1990). Following *Diathoneura*, the next split in Drosophilinae leads to *Scaptodrosophila latifasciaeformis*, and, after that, to two major clades: (i) *Colocasiomyia*, *Chymomyza*, *Neotanygastrella*, and *Scaptodrosophila lebanonensis* and (ii) *Drosophila sensu lato* and *Scaptodrosophila inornata*.

The first of these clades corroborates the results of Yassin (2013), but the relationships among taxa within this clade must be seen as weakly supported, due to the topological discordance among species trees inference analyses and low concordance factors (Fig. 1). The second clade includes taxa within *Drosophila sensu lato*, a well-known nonmonophyletic assemblage that also comprises *Zygothrica*, *Zaprionus*, and *Scaptomyza*. The relationships within *Drosophila sensu lato* support those recovered in recent phylogenomic studies (eg Suvorov et al. 2022; Kim et al. 2024)

*Scaptodrosophila* was not recovered as a monophyletic lineage, echoing prior findings (eg van der Linde et al. 2010; Russo et al. 2013; Finet et al. 2021; Kim et al. 2024). This result was supported in all our analyses, regardless of the method used to infer the species tree (Figs. 1 and S1 to S18). Likelihood-based topology tests comparing the unconstrained ML tree (Fig. 1) with a constrained tree enforcing *Scaptodrosophila* monophyly showed that the constrained tree was significantly less likely (Kishino-Hasegawa test: *P* = 0; Shimoidara-Hasegawa test: *P* = 0; Approximately Unbiased test: *P* = 2.16 × 10^−6^), thereby rejecting the monophyly of *Scaptodrosophila* in our dataset. These findings support earlier suggestions (O’Grady and DeSalle 2018) that the genus may need to be split.

A comparison of our topology with previous studies is summarized in Figs. S21 and S22, and a summary of tribe-level classification results is provided in Table S2 and further discussed in File S1. Taken together, our results underscore the complexity of early divergences within Drosophilidae and the limitations of the historically proposed tribes when mapped onto phylogenomic data. While some deep clades appear robust and repeatable across studies, others remain unstable. Continued taxon sampling and integration of morphological character sets will be essential to refining the evolutionary narrative of the group.

### Gene Tree Heterogeneity and Uncertain Relationships

Despite the use of a large dataset (1,606 genes and over 2.4 million sites), some relationships within the Drosophilinae subfamily remained unresolved. Three observations support this uncertainty: (i) reduced bootstrap values in several nodes, (ii) low gCF and sCF values, and (iii) instability in the placement of certain lineages across inference methods (Figs. 1 and S1 to S18). It is noteworthy that these problems also occur with protein-based analyses (Figs. S11 and S18), which are less prone to homoplasy and variation in base composition across species.

While bootstrap values typically increase with dataset size, several nodes in our tree showed support <90%, indicating nontrivial discordance. These nodes also exhibited markedly low gCF and sCF, suggesting limited phylogenetic signal or underlying conflict due to biological processes. For example, the branch uniting *Chymomyza* and *Neotanygastrella* showed a gCF <33%, falling into the parameter space where ILS can mislead concatenated analyses (Kubatko and Degnan 2007).

Such discordance is likely influenced not only by ILS but also by introgression. Gene tree heterogeneity has been previously documented in *Drosophila* and other Diptera (eg Pollard et al. 2006; Araki et al. 2013; Dias et al. 2020; Suvorov et al. 2022), with introgression proposed as a contributing factor. To explore this hypothesis, we used PhyloNet, a network-based inference method allowing for reticulation events (Wen et al. 2018). Our analysis (see File S2) identified three candidate introgression events involving the ancestors of *Cryptochetum*, Drosophilinae, and *Drosophila*, including two unsampled “ghost” lineages. While these events did not clarify major topological conflicts (eg the placement of *Scaptodrosophila*), they support the role of horizontal gene flow in early Drosophilidae diversification.

It is worth noting that the main topology presented in this study was inferred via ML concatenation using the first and second codon positions of the comprehensive dataset (Fig. 1). While this method is known to be inconsistent in the presence of ILS and introgression (Kubatko and Degnan 2007), our ASTRAL analyses, which rely on the multispecies coalescent model, yielded broadly similar results, including the same unstable placements of “rogue” taxa such as *Scaptodrosophila* (Figs. S3 to S4, S8 to S11, and S15 to S18). Thus, despite methodological differences, both approaches converge on the conclusion that some evolutionary relationships remain difficult to resolve—likely due to a combination of ILS, introgression, and limited phylogenetic signal.

### Timing of Drosophilidae Early Radiation

**Fig. 2.**
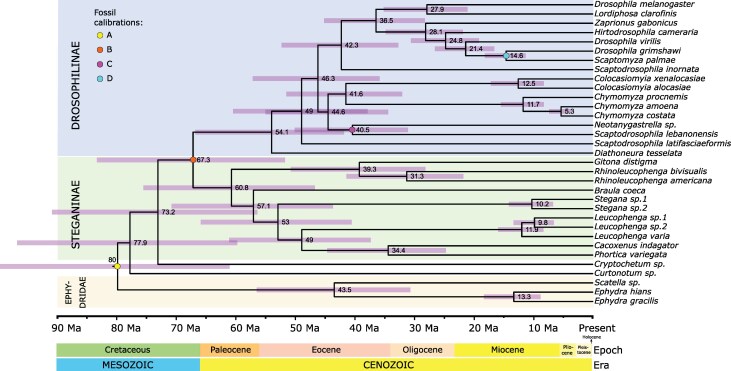
Divergence time inference for Drosophilidae obtained with MCMCTree. Purple bars represent the 95% HPD intervals, and mean posterior divergence times are shown near the corresponding nodes. The outgroup and the subfamilies Steganinae and Drosophilinae are highlighted in orange, green, and blue, respectively. Calibration points are shown as colored dots on specific nodes and labeled A to D, matching the labels used in the text for ease of reference. This analysis was based on the ML tree from the “comprehensive” dataset using first and second codon positions (Fig. 1).

Fossil scarcity and limited genomic coverage have long hindered precise dating of Drosophilidae diversification. Here, we used a genome-scale dataset combined with fossil calibrations to estimate divergence times across major drosophilid lineages (Fig. 2; Table 2; see Materials and Methods and File S3 for calibration details). Our priors are conservative (see Materials and Methods) and it would be undesirable if they unduly influenced the estimated divergence times. Yet, we found that the divergence time estimates did gain information from the data. In all cases, the 95% highest posterior density (HPD) intervals were narrower and shifted relative to the effective priors, indicating that our divergence time estimates were primarily informed by the molecular data, and not by the priors. For example, the effective prior for the most recent ancestor (MRCA) of Ephydroidea spanned 114.6 to 40.0 Ma, whereas the posterior was narrowed to 99.8 to 61.1 Ma. Similar patterns were observed for all calibrated nodes (Fig. S23).

We inferred that Ephydroidea began diversifying around 80 Ma (95% HPD: 100 to 61 Ma), and that Drosophilidae originated near the Cretaceous–Paleogene (K–Pg) boundary, at ∼67.3 Ma (95% HPD: 83.5 to 51.8 Ma). This is broadly consistent with previous studies (eg Wiegmann et al. 2011; Bayless et al. 2021; Suvorov et al. 2022) and supports a Mesozoic origin for the family. Although the oldest known fossil definitively assigned to Drosophilidae (*Electrophortica succini*) is from the Late Eocene, molecular estimates—including ours—point to a much earlier origin.

Compared to Suvorov et al. (2022), our time estimates are similar for deep nodes (eg Drosophilidae MRCA) but younger for shallow splits (eg *Scaptomyza–Drosophila virilis*). Moreover, our posterior distributions presented wider 95% HPD intervals. These differences may result from our broader representation of non-*Drosophila* taxa and more conservative prior specifications (see File S3). Importantly, both studies employed MCMCTree, which does not account for gene tree heterogeneity and may underestimate divergence times in the presence of gene flow (Tiley et al. 2023). A multispecies-coalescent-with-introgression method (Tiley et al. 2023) was not employed because of the high computational demands, which makes this analysis impractical for our dataset.

We estimated the most recent common ancestors (MRCAs) of Steganinae and Drosophilinae at 60.8 Ma (95% HPD: 75.6 to 46.8 Ma) and 52 Ma (66.9 to 41.9 Ma), respectively. Most divergences leading to extant genera occurred during the Eocene (56 to 34 Ma), a period of increased insect diversification (Labandeira and Sepkoski 1993; Jarzembowski and Ross 1996; Dmitriev et al. 2018). The split between the subgenera *Drosophila* and *Sophophora* was dated at ∼36 Ma (45.2 to 28.2 Ma), slightly younger than estimates based on island biogeography (eg Tamura et al. 2004; Russo et al. 2013) but close to those derived from empirically estimated mutation rates and revised Hawaiian colonization models (Obbard et al. 2012).

Within Steganinae, the MRCA of *Leucophenga* and *Stegana* was estimated at 53 Ma (95% HPD: 64.7 to 39.0 Ma), aligning closely with Li et al. (2013). The divergence of *B. coeca* from its closest relatives was dated at 57.1 Ma (70.9 to 43.7 Ma), suggesting an earlier origin of the *Braula* lineage than proposed by Bastide et al. (2024), although both estimates substantially overlap. This result reinforces the view that *Braula*'s host association with Apidae emerged in the Paleocene or early Eocene.

### Substitution Rates in Drosophilidae

We estimated substitution rates separately for first + second and third codon positions. As expected, the rate for first and second codon positions was much lower (3.3 substitutions/kb/My, 95% HPD: 2.5 to 4.1 substitutions/kb/My) than that of the third codon position (16.7 substitutions/kb/My, 95% HPD: 13.1 to 21.1 substitutions/kb/My), reflecting much stronger purifying selection at nonsynonymous sites. Importantly, these estimates are based directly on genomic data and do not rely on generation times.

The third codon position estimate (16.7 substitutions/kb/My) serves as a proxy for the neutral evolutionary rate and is lower than estimates derived from *Drosophila* mutation rates based on mutation accumulation lines and parent–offspring sequencing (Keightley et al. 2009, 2014; Schrider et al. 2013; Wang et al. 2023). Because mutation rates are per-generation, to obtain an approximation of the neutral evolutionary rate, they must be converted to geological time using assumptions about the number of generations per year. Assuming 10 generations per year (Cutter 2008; Obbard et al. 2012), we get 28 to 55 substitutions/kb/My; assuming 15 generations per year (Pool 2015), they rise to 42 to 83 substitutions/kb/My. However, generation times in Drosophilidae can be markedly longer, particularly in environments where reproduction is seasonally constrained and diapause limits populations to only a few generations per year (Schmidt et al. 2005; Mitsui et al. 2010; Behrman et al. 2015). This reinforces the value of fossil-calibrated analysis using third codon positions as a proxy for the neutral evolutionary rates, as this analysis is agnostic to generation time assumptions.

Nevertheless, like all molecular dating approaches, our method depends on other assumptions (eg fossil calibration accuracy). Yet, our estimate matches the estimate of 19.0 substitutions/kb/My reported by Obbard et al. (2012), obtained under a Bayesian relaxed clock model assuming colonization of the Hawaiian Islands after shield completion and a very small ancestral population size. In fact, the 95% HPD of our estimate (13.1 to 21.1 substitutions/kb/My) encompasses a rate of 19.0 substitutions/kb/My. This convergence suggests that, despite methodological differences, both estimates reflect similar patterns of long-term neutral evolution in Drosophilidae. In contrast, these rates are higher than estimates from Tamura et al. (2004) and Russo et al. (1995), which used a limited number of genes and relied on the emergence of Kauai Island (∼5.1 Ma) as a calibration point, rather than colonization after shield completion.

A comparative overview of substitution rate estimates, along with methodological and calibration details, is provided in Table 3.

**Table 3.** Comparisons of the substitution rates estimated for drosophilids

Study	Estimated rate	Estimation method	Events/kb/My
Keightley et al. (2009) ^a^	SNM	DNA sequencing (MA lines)	34.6
Keightley et al. (2014) ^a^	SNM	DNA sequencing (parent–offspring)	28.0
Wang et al. (2023) ^a^	Mean SNM rate across *D. melanogaster* and *D. simulans*	DNA sequencing (parent–offspring)	33
Schrider et al. (2013) ^a^	Average SNM rate across two inbred lines	DNA sequencing (MA lines)	55
Keightley et al. (2009) ^b^	SNM	DNA sequencing (MA lines)	51.9
Keightley et al. (2014) ^b^	SNM	DNA sequencing (parent–offspring)	42
Wang et al. (2023) ^b^	Mean SNM rate across *D. melanogaster* and *D. simulans*	DNA sequencing (parent–offspring)	49.5
Schrider et al. (2013) ^b^	Average SNM rate across two inbred lines	DNA sequencing (MA lines)	82.5
Obbard et al. (2012)	3rd codon pos. substitution	Biogeography^c^, small population	8.0
Obbard et al. (2012)	3rd codon pos. substitution	Biogeography^c^, large population	6.0
Obbard et al. (2012)	3rd codon pos. substitution	Biogeography^d^, small population	19.0
Obbard et al. (2012)	3rd codon pos. substitution	Biogeography^d^, large population	10.0
Tamura et al. (2004)	4-fold degenerate distance	Biogeography^e^	11.0
Russo et al. 1995	3rd codon pos. substitution	Biogeography^e^	10.0
This study	1st and 2nd codon pos. substitution	Fossil calibration	3.3
This study	3rd codon pos. substitution	Fossil calibration	16.7

SNM, single-nucleotide mutation.

^a^Converted to a time-based estimate assuming ten generations per year (based on Cutter [2008]; Obbard et al. [2012]).

^b^Converted to a time-based estimate assuming 15 generations per year (based on Pool [2015].

^c^Hawaiian Islands emergence.

^d^Hawaiian Islands shield completion.

^e^Formation of Kauai Island.

## Conclusions

Conflicting results have marked the field of Drosophilidae phylogenetics. Here, we demonstrate that much of this conflict arises from extensive gene tree heterogeneity driven by ILS and introgression, which complicates resolution with traditional methods. While we recovered the monophyly of Drosophilinae for the sampled taxa, the placement of *B. coeca* within Steganinae rendered Drosophilidae nonmonophyletic under our framework. Furthermore, our results do not support the monophyly of most tribes previously proposed for either subfamily, reinforcing the need for a broad taxonomic reevaluation.

Despite recent advances in genomic coverage across the family (eg Kim et al. 2024), early-diverging and taxonomically unstable lineages remain underrepresented in most phylogenomic studies. Our study addressed this gap by focusing on these lineages; yet, key branches—such as those involving *Scaptodrosophila*, *Chymomyza*, and *Neotanygastrella* lineages—remain unstable. Broader sampling within non-*Drosophila* lineages will be essential for resolving these relationships.

Our fossil-calibrated divergence time estimates suggest that Drosophilidae originated near the Cretaceous–Paleogene boundary (∼67 Ma), with most extant genera arising during the Eocene (56 to 34 Ma). While credible intervals around these estimates remain wide, they are consistent with other fossil-based and molecular studies. These temporal estimates provide a framework to understand drosophilid evolutionary history in the context of major geological and climatic events. Improving the fossil record of the group—and integrating methods that better account for introgression—will be key to refining the temporal framework of drosophilid diversification.

## Materials and Methods

### Species Sampling

We used molecular sequences of 33 Ephydroidea species, including 27 traditionally classified as Drosophilidae—10 from the Steganinae subfamily and 17 from the Drosophilinae subfamily (Table S2). The remaining species belong to other Ephydroidea families: *B. coeca* (belonging to Braulidae), *Cryptochetum* sp. (Cryptochetidae), *Curtonotum* sp. (Curtonotidae), and *Ephydra gracilis*, *Ephydra hians*, and *Scatella* sp. (Ephydridae, which is believed to be the least closely related to Drosophilidae and thus used as outgroup; Wiegmann et al. 2011; O’Grady and DeSalle 2018; Bayless et al. 2021). *Drosophila* is currently known to be paraphyletic to several other genera, such as *Hirtodrosophila, Scaptomyza*, *Zaprionus*, and *Mycodrosophila* (O’Grady and DeSalle 2018). Yet, since our focus was the early radiations in Drosophilidae, we used only seven species from the *Drosophila* sensu lato group (namely, *D. virilis, Drosophila melanogaster*, *Drosophila grimshawi*, *Lordiphosa clarofinis*, *Hirtodrosophila cameraria*, *Zaprionus gabonicus*, and *Scaptomyza palmae*). The other ten Drosophilinae are external to *Drosophila* and belong to five genera: *Scaptodrosophila*, *Colocasiomyia*, *Neotanygastrella*, *Chymomyza*, and *Diathoneura*.

Genome sequencing data were used for 30 of the 33 species, with 16 obtained from NCBI and 14 sequenced for the present work. For the remaining three species—*Curtonotum* sp., *Cryptochetum* sp., and *B. coeca*—transcriptomic data were downloaded from NCBI (https://www.ncbi.nlm.nih.gov). Details about the sources of all sequences are provided in Table S3 and File S4.

The specimens used for genomic sequencing were identified based on diagnostic morphological characters following widely used taxonomic keys (McAlpine et al. 1981; Vilela and Bächli 1990; Bächli et al. 2004; Brown et al. 2010, see File S4 for more information on collection and identification). For *Diathoneura tessellata*, taxonomic confirmation and ecological information were based on Collier and Armstrong (2009). Some specimens could not be identified to the species level (eg *Stegana*, *Leucophenga*, *Neotanygastrella*, and *Scatella*) or had uncertain specific identification (eg *Rhinoleucophenga cf. bivisualis*). However, this is not problematic, as this study primarily focuses on relationships among genera, tribes, and subfamilies. For *R. cf. bivisualis,* we considered the possibility that the specimens collected in the Brazilian Savanna (Cerrado) and identified as *R. bivisualis* might actually belong to *Rhinoleucophenga punctulata*, as noted by Vilela and Bächli (2009). A thorough discussion on this issue is provided in File S4.

### Genomes and Transcriptomes Sequencing and Assembling

*D. melanogaster* and *D. virilis* genomes were previously sequenced with Sanger technology and assembled with the appropriate assemblers (Adams et al. 2000; Clark et al. 2007; Hoskins et al. 2015). *Phortica variegata, S. lebanonensis, E. hians*, and *E. gracilis* genomes were sequenced with Illumina technology by Vicoso and Bachtrog (2015). *Cacoxenus indagator, Chymomyza amoena, Colocasiomyia xenalocasiae*, and *R. cf. bivisualis* genomes were sequenced by our group as described in Dias et al. (2020). *Chymomyza costata*, *Leucophenga varia*, *Z. gabonicus*, *L. clarofinis*, and *D. grimshawi* were sequenced with Nanopore technology and assembled by Kim et al. (2021). The *Hirtodrosophila cameraria* assembly is based on 45× PacBio and Arima2 Hi-C data provided by the Darwin Tree of Life Project (Obbard 2023). Finally, the transcriptome of *B. coeca* originates from the 1,000 Insect Transcriptome Evolution Project (1KITE), and the transcriptomes of *Curtonotum* sp. and *Cryptochetum* sp. from Bayless et al. (2021). The source of all raw reads libraries and assemblies are presented in Table S3.

We sequenced the remaining 14 species as follows. We extracted DNA from one individual or a pool of flies preserved in −20 °C ethanol using the Puregene DNA kit (QIAGEN) and following the manufacturer's recommendations. For specimens not identified to the species level or belonging to species with unavailable laboratory strains, we extracted DNA using a nondestructive method that preserves the exoskeleton (Santos et al. 2018). This approach preserves the flies’ morphology, allowing for future studies. Illumina sequencing (350 bp fragment size, paired-end libraries, 150 bp read size) was carried out at Macrogen (Korea) with HiSeq 2000.

The genomes were assembled using SPAdes v. 3.9.0 or 3.11.1 (Bankevich et al. 2012) and Platanus 1.2.4 (Kajitani et al. 2014). BlobTools (Laetsch et al. 2017) was used to screen assemblies for contaminants, and QUAST (Gurevich et al. 2013), GenomeScope (Vurture et al. 2017), and BUSCO v3 (Simão et al. 2015) were used to assess the quality of assemblies (Tables S4 and S5).

### Gene Annotation and Ortholog Identification

We followed a protocol similar to that described in Dias et al. (2020) for identifying orthologs and annotating genes. After assembling the genomes, we used BUSCO v3 (Simão et al. 2015) to assess the assemblies’ quality and to annotate single-copy orthologs. BUSCO searched for 2,799 universal single-copy orthologs from the Diptera reference set of orthologs (odb9, downloaded in https://busco.ezlab.org/on 2018 March 22) in our 33 assemblies, reporting how many of them were complete, duplicated, fragmented, or missing in each species (Table S5). Since BUSCO annotates single-copy orthologs along this process, the resulting sequences are suitable for phylogenomic inference (Waterhouse et al. 2018).

We used the script fix_busco_CDS_frame.awk (Dias et al. 2020) to correct the frame of BUSCO annotated sequences and removed individual sequences that had a coefficient of variation of protein size larger than 10%. Then, we gathered three datasets of orthologs, hereafter referred to as the “conservative,” “relaxed,” and “comprehensive” datasets. These datasets differ in both taxon sampling and gene inclusion thresholds. The “conservative” and “relaxed” datasets included 24 species selected to optimize taxon representation while allowing faster analyses. Species were excluded based on three criteria: (i) species from genera represented by at least two other species (*L. varia* and *C. costata*); (ii) species within *Drosophila* sensu lato, as their internal relationships fall outside the scope of this study (*Z. gabonicus*, *L. clarofinis*, *H. cameraria*, and *D. grimshawi*); and (iii) species for which transcriptomic sequences were used (*B. coeca*, *Curtonotum* sp., and *Cryptochetum* sp.).

The “conservative” dataset includes 448 genes present in all 24 species, while the “relaxed” dataset includes 1,252 genes present in at least 22 of the 24 species. Lastly, the “comprehensive” dataset, which forms the basis of the main analyses discussed in this paper, includes all 33 species and comprises genes present in at least 25 taxa, yielding 1,606 genes.

### Phylogenetic Inference and Molecular Dating

We used the Perl script translatorx_vLocal.pl (Abascal et al. 2010) to align nucleotide sequences based on their translated amino acid sequences, using MAFFT v7.394 as the aligner (-p F -g 1). Then, we inferred ML phylogenetic trees for each gene using best-fit models in IQ-TREE (Nguyen et al. 2015; Kalyaanamoorthy et al. 2017). Finally, we used TreeShrink in “per-species” mode to identify and remove other outliers in gene trees (Mai and Mirarab 2018).

We obtained the GC content of the first + second codon positions (GC12) and of the third codon positions (GC3) from all sequences of the filtered “comprehensive” dataset (1,606 genes across 33 taxa). We tested the statistical significance of the species heterogeneity in GC12 and GC3 with a one-way ANOVA in SYSTAT (Wilkinson 2010).

To infer species trees, we performed 18 distinct phylogenetic analyses using the three datasets of orthologs (“comprehensive,” “relaxed,” and “conservative”). The comprehensive dataset was used for the main phylogenetic inferences discussed in the text, while the relaxed and conservative datasets were used for supplementary analyses to test the robustness of the results under different taxon samplings and gene inclusion thresholds.

We applied a combination of concatenation-based ML and MSC approaches to each dataset. For the comprehensive dataset, we performed four analyses: two based on concatenated nucleotide alignments (partitioned by gene, using either all codon positions or only first and second codon positions), and two based on the MSC framework—one using gene trees inferred from all codon positions, and another using only first and second codon positions. Concatenated analyses were performed in IQ-TREE (Nguyen et al. 2015). The best-fit model for each partition was selected using ModelFinder (Kalyaanamoorthy et al. 2017). Branch support was assessed via 1,000 ultrafast bootstrap replicates, and we computed gCFs and sCFs to evaluate support and conflict among loci (Minh et al. 2020). MSC analyses were conducted using ASTRAL-III (Mirarab et al. 2014), using local posterior probabilities to assess support.

For the relaxed and conservative datasets, we conducted seven analyses each. Concatenated analyses employed three partitioning strategies: (i) by gene, (ii) by codon position (two partitions: first + second and third), and (iii) by gene using only first and second codon positions. In each case, the same procedure as described above was performed to select the best-fit model for each partition an assess branch supports.

For MSC-based analyses, we used ASTRAL-III to infer species trees from sets of gene trees, and assessed support with local posterior probabilities. Gene trees used in these analyses were inferred from alignments based on four alternative data types: (iv) all codon positions, (v) only first and second codon positions, (vi) all codon positions and the GHOST model (Crotty et al. 2020), which accounts for heterotachy (ie variations in lineage-specific evolutionary rates over time), and (vii) translated sequences (amino acids).

Table 1 summarizes the 18 analyses and provides additional information about them. Representative fly images used in Fig. 1 were generated from reference photographs using ChatGPT (OpenAI), a generative artificial intelligence tool. These images were produced exclusively for illustrative purposes and did not influence data analysis or interpretation.

To detect introgression events, we inferred phylogenetic networks using PhyloNet (Wen et al. 2018). This analysis was performed under maximum pseudo-likelihood and was based on gene trees derived from the “relaxed” dataset. To decide how many reticulations to use, we followed the empirical approach described in Hibbins and Hahn (2022), which resulted in three reticulation events. See File S2 for a thorough discussion on PhyloNet methods and results.

To establish a Drosophilidae timescale, we inferred node ages based on the “comprehensive” dataset using two partitions—one for the first and second codon positions and one for the third codon positions (Fig. 2). We inferred Bayesian time-trees using the software MCMCTree (dos Reis and Yang 2011) from the PAML v 4.9 package (Yang 2007) and a relaxed clock with rate variation among sites. This analysis requires a fixed species tree (we used the tree topology obtained in analysis 2—ie obtained in IQTREE by concatenated first and second codon positions) and a prior for the overall rate parameter, which was derived from a codeml analysis under the global clock model with point calibrations (rgene_gamma = 1.15443 2). The rate drift parameter was set as “sigma2_gamma = 1 1” and the parameters of the birth-death process were “BDparas = 1 1 0”. The time unit was set to be 100 My. We applied an independent rates model (clock = 2) and the general time-reversible substitution model (Tavaré 1986). Two independent trials of the Markov chain Monte Carlo (MCMC) were carried out with 700,000,000 generations each and a sampling frequency of 1,000 (burn-in = 1,000,000). effective sample size values were higher than 200 for all MCMC parameters and the slope and *R*^2^ of the linear regression between the posterior mean estimates derived from the two runs were 1. MCMCTree was also run with usedata = 0, sampling from the prior, in order to compare the effective prior and posterior distributions (Fig. S23).

### Fossil Information and Node Calibration

We followed the protocol of best practices suggested by Parham et al. (2012) to select fossils for node calibration. We considered all well-identified fossils available and reviewed their proposed phylogenetic placements using apomorphy-based diagnosis. Additionally, we reviewed the published geological ages and stratigraphic ranges of the fossils and synchronized these data with a standard global geochronology retrieved from the Paleobiology Database, accessed on 2020 June 21 via the Fossilworks gateway (Alroy 2020). The ages of geological periods follows the International Chronostratigraphic Chart v 2019/5 (Cohen et al. 2013). All fossils considered for calibration are summarized in Table S6.

As the fossil record of Drosophilidae is scarce, we were able to retrieve only 15 fossils (Cockerell 1923; Statz 1940; Hennig 1965; Grimaldi 1987, 1993; Alroy 2020), of which only three met our criteria (secure phylogeny, nonredundancy, reliable age): *E. succini* (Hennig 1965), *Neotanygastrella wheeleri* (Grimaldi 1987), and *Scaptomyza dominicana* (Grimaldi 1987). These fossils were used to calibrate four nodes with skew-normal prior distributions that set fossil ages as minimum bounds while incorporating evidence-based soft maxima to avoid arbitrary truncation.

For the Ephydroidea MRCA (Node A, Fig. 2), we used the oldest Drosophilidae fossil, the Steganinae *E. succini* (37.2 to 33.9 Ma), from Danish Baltic amber, with a skew-normal prior (*ξ* = 0. 378, *ω* = 0.35, *α* = 10). The 95% cumulative probability interval for this distribution ranged from 116.2 to 37.2 Ma. Therefore, 116.2 Ma was used as a soft maximum that integrates: (i) the hard constraint from the oldest Cyclorrhapha fossil (*Opetiala shatalkini*; 145 to 140 Ma; Grimaldi and Engel 2005); (ii) the mid-Cretaceous insect turnover (∼100 Ma; Zherikhin 2002; Dmitriev et al. 2018) as an earliest plausible diversification window; and (iii) fossil-calibrated estimates for nested clades like the Drosophilidae MRCA ≤85.9 Ma (HPD upper bound estimated in Russo et al. 2013), which preclude substantially older origins.

The same *E. succini* fossil was used to calibrate the Drosophilidae MRCA (Node B, Fig. 2) under a distinct skew-normal prior (*ξ* = 0.343, *ω* = 0.20, *α* = 10; 95%), for which the 95% cumulative probability boundaries ranged from 79.1 to 33.9 Ma. The 79.1 Ma soft maximum accommodates the HPD upper bound of published fossil-calibrated estimates (Russo et al. 2013; Suvorov et al. 2022).

Using the minimum age of *N. wheeleri* (23.03 to 15.97 Ma; Chiapas amber), we calibrated the MRCA of *Neotanygastrella* sp. and *S. lebanonensis* (Node C, Fig. 2) with a skew-normal prior (*ξ* = 0.1633, *ω* = 0.20, *α* = 10), with the 95% cumulative probability interval spanning 61.2 to 15.97 Ma. This broad calibration prior reflects the uncertainty surrounding the age of this split, as this is the first study to evaluate it, and allows for the possibility that it occurred as far back as the K–Pg boundary, where recent molecular dating studies place the origin of crown-Drosophilidae (Suvorov et al. 2022).

Finally, the minimum age of *S. dominicana* (20.43 to 13.65 Ma; Dominican amber) calibrated the *D. grimshawi–S. palmae* divergence (Node D, Fig. 2) under a skew-normal prior (*ξ* = 0.1392, *ω* = 0.15, *α* = 10). The 95% cumulative probability interval ranged from 47.5 to 13.65 Ma, with most prior density concentrated near the fossil age. The soft maximum is conservative, extending above published estimates for this split—29.6 to 21.9 Ma in Russo et al. (2013) and roughly 30 to 15 Ma in Suvorov et al. (2022)—so as not to overconstrain divergence time inference while still remaining consistent with available evidence.

## Supplementary Material

msaf269_Supplementary_Data

## Data Availability

The new SRA and genome assembly data underlying this article are available in NCBI GenBank at https://www.ncbi.nlm.nih.gov/sra/PRJNA929699 and can be accessed with PRJNA929699. Alignment files are available at https://github.com/GuilhermeRDias/Drosophilidae_phylogenomics_2.
