# Supplementary material for: Timing and Pattern of Early Diversification in Drosophilidae (Diptera)
Source: Mol Biol Evol. 2025 Oct 23;42(11):msaf269. doi: 10.1093/molbev/msaf269 (PMC12603360; doi:10.1093/molbev/msaf269)
Supplement: msaf269_Supplementary_Data [file msaf269_supplementary_data.zip › MBE25-0386_Dias_Supplementary_files.pdf]

Supplementary file 1: Tribe-Level Relationships in Drosophilidae

The classification of Drosophilidae in tribes has historically relied on morphology (*e.g.*, Okada 1989; Grimaldi 1990), with later revisions incorporating limited molecular evidence (*e.g.*, Yassin 2013). However, the monophyly and composition of many tribes remain debated. Here, we use our phylogenomic dataset to test whether currently proposed tribes are supported by genome-scale evidence. Table S2 summarizes the tribes represented in our dataset, the sampled genera, and whether their monophyly was recovered.

Table S2. Summary of whether each tribe proposed in the literature (Okada 1989; Grimaldi 1990; Sidorenko 2002; Yassin 2013) for Drosophilidae was recovered as monophyletic in our dataset. Tribes represented by a single genus were not formally tested for monophyly.

Subfamily	Tribe (Original Author)	Monophyly Recovered
**Drosophilinae**	Drosophilini (Okada, 1989)	No
	Drosophilini (Grimaldi, 1990)	No
	Drosophilini (Yassin, 2013)	Yes
	Colocasiomyini (Okada, 1989)	Not tested (1 genus)
	Colocasiomyini (Yassin, 2013)	Yes
	Cladochaetini (Grimaldi, 1990)	Not tested (1 genus)
	Microdrosophilini (Okada, 1989)	Not sampled
	Hypselothyrini (Okada, 1989)	Not sampled
	Dettopsomyini (Okada, 1989)	Not sampled
**Steganinae**	Steganini (Okada, 1989)	No
	Leucophengini (Okada, 1989)	No
	Steganini (Sidorenko, 2002)	No
	Gitonini (Sidorenko, 2002)	No
	Steganini (Grimaldi, 1990)	No
	Gitonini (Grimaldi, 1990)	No
	Steganini (Yassin, 2013)	No
	Gitonini (Yassin, 2013)	No

Drosophilinae

Our sampling includes representatives from nearly all proposed tribes, except for Microdrosophilini, Hypselothyrini, and Dettopsomyini (Okada 1989). These three were included in Kim et al. (2024), where all fell within the *Drosophila sensu lato* clade.

In our data, neither Drosophilini sensu Okada (1989) nor sensu Grimaldi (1990) were supported. In contrast, Drosophilini sensu Yassin (2013)—which includes *Drosophila*, *Zaprionus*, and *Scaptomyza*—was recovered as monophyletic in our analyses. For tribes represented by only one genus (*e.g.*, Colocasiomyini sensu Okada (1989) and Cladochaetini sensu Grimaldi (1990)), monophyly could not be assessed.

Diathoneura consistently emerged as the earliest diverging lineage within Drosophilinae, supporting its inclusion in the family (Grimaldi 1990) and contradicting Yassin's (2013) proposal to reassign it to Ephydridae. Our topology thus supports its retention in Cladochaetini sensu Grimaldi (1990). After *Diathoneura*, *Scaptodrosophila latifasciaeformis* was consistently inferred as the next diverging lineage, followed by two major clades:

(1) *Colocasiomyia*, *Chymomyza*, *Neotanygastrella*, and *Scaptodrosophila lebanonensis*, and

(2) *Scaptodrosophila inornata* and *Drosophila sensu lato*.

This pattern broadly aligns with the redefinition of Colocasiomyini by Yassin (2013), which groups together non-crown Drosophilinae outside the *Drosophila* clade. However, support for internal relationships within these clades was generally low, with high topological discordance and low concordance factors across species trees (see Fig. 1).

Steganinae

The subfamily Steganinae has been historically divided into two or more tribes, but their definitions vary across authors. Okada (1989) proposed Steganini (including *Stegana*, *Gitona*, *Cacoxenus*, and *Phortica* as part of *Amiota*) and Leucophengini (including *Leucophenga* and *Rhinoleucophenga*). Grimaldi (1990) proposed a different structure,

assigning *Stegana* and *Leucophenga* to Steganini, and *Rhinoleucophenga*, *Gitona*, *Cacoxenus*, and *Phortica* to Gitonini. Yassin (2013) retained the same tribe names but altered genus compositions again, grouping *Leucophenga* and *Stegana* in Steganini, and *Phortica*, *Cacoxenus*, *Gitona*, and *Rhinoleucophenga* in Gitonini. Finally, Sidorenko (2002) proposed tribes Steganini (grouping *Stegana* and *Leucophenga*) and Gitonini (including *Gitona*, *Phortica* and *Cacoxenus*).

None of these tribes were recovered as monophyletic in our analyses. Our topology consistently revealed two main clades within Steganinae: (i) one containing *Rhinoleucophenga* and *Gitona*, and (ii) another grouping *Braula*, *Stegana*, *Leucophenga*, *Phortica*, and *Cacoxenus*. These results are congruent with other recent phylogenomic analyses (Bastide et al. 2024; Kim et al. 2024).

Summary

Overall, our analyses do not support the monophyly of most traditional tribes of Drosophilidae, wheter in Drosophilinae or Steganinae (*e.g.*, Okada 1989; Grimaldi 1990; Yassin 2013). Only Drosophilini and Colocasiomyini sensu Yassin (2013) were recovered, and even then, internal support varied. Several tribes remain untestable due to sparse sampling, and others—particularly those containing problematic genera like *Scaptodrosophila*—require formal taxonomic revision. Our results suggest that a comprehensive reevaluation of tribe-level classification in Drosophilidae is both timely and necessary.

References:

Bastide H, Legout H, Dogbo N, Ogereau D, Prediger C, Carcaud J, Filée J, Garnery L,

Gilbert C, Marion-Poll F, et al. 2024. The genome of the blind bee louse fly reveals deep convergences with its social host and illuminates Drosophila origins. Curr. Biol. 34:1122-1132.e5.

Grimaldi DA. 1990. A phylogenetic, revised classification of genera in the Drosophilidae (Diptera). Bull. Am. Museum Nat. Hist. 197:1–139.

Kim BY, Gellert HR, Church SH, Suvorov A, Anderson SS, Barmina O, Beskid SG, Comeault AA, Crown KN, Diamond SE, et al. 2024. Single-fly genome assemblies fill major phylogenomic gaps across the Drosophilidae Tree of Life. PLoS Biol. [Internet] 22:1–23. Available from: http://dx.doi.org/10.1371/journal.pbio.3002697

Okada T. 1989. A Proposal of Establishing Tribes for the Family Drosophilidae with Key to Tribes and Genera (Diptera) : Taxonomy and Systematics. Zoolog. Sci. 6:391–399.

Sidorenko VS. 2002. Phylogeny of the tribe Steganini Hendel and some related taxa (Diptera, Drosophilidae). Far East. Entomol. 111:1–20.

Yassin A. 2013. Phylogenetic classification of the Drosophilidae Rondani (Diptera): the role of morphology in the postgenomic era. Syst. Entomol. 38:349–364.

Supplementary file 2: Phylonet analyses

We used PhyloNet to investigate signals of introgression across the Ephydroidea phylogeny. This software compare network topologies and use a single heuristic for reconciling gene trees with a species tree, producing phylogenetic networks that extent the tree model to allow horizontal edges which represent gene flow among taxa (Wen et al. 2018).

We used the maximum pseudo-likelihood (MPL) approach to infer phylogenetic networks. To do this, we had to evaluate how many reticulation events we would allow in our analysis. A parameter to evaluate this is the "total log probability" of the inferred networks. However, there is an issue with this approach: more complicated graphs assign better likelihoods. Thus, networks allowing for more reticulations tend to have better "total log probability" scores.

To decide how many reticulations we should use, we followed an empirical approach described in Hibbins & Hahn (2022): we used "a slope heuristic where networks are inferred across different numbers of reticulations, and the best network is taken as the least complex one after which the likelihood score appears to stop improving" (Hibbins and Hahn 2022). For doing so, we inferred 30 networks, five for each number of reticulations, from 0 to 5. Then we plotted the "total log probabilities" of each network, to see when the inclusion of additional reticulations stops improving it. This occurs with 3 reticulations (Fig.1).

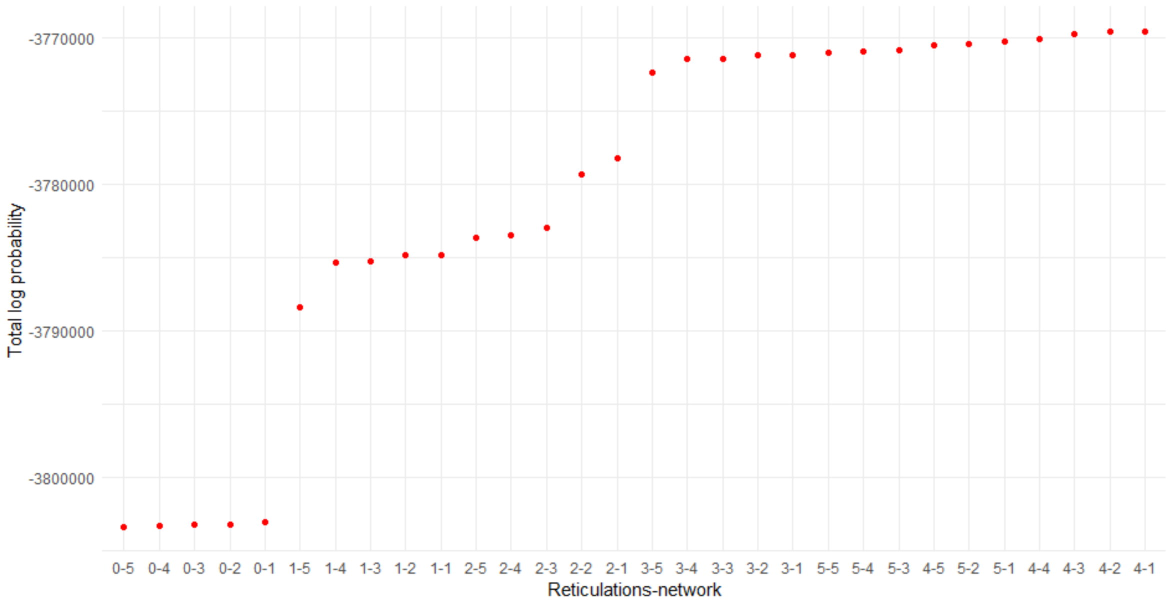

Fig. 1. Total log probabilities of phylogenetic networks inferred in PhyloNet, from the lowest (left) to the highest (right). The number codes on the x-axis refer, respectively, to the number of reticulations and the number of the network (*e.g.* "0-5" means the 5th network generated on the analysis allowing for 0 reticulations).

Then, we used the PhyloNetworks Julia package (Solís-Lemus et al. 2017) to visualize the networks with 3 reticulations (Fig. 2). We also used Dendroscope and Icytree but decided to prioritize PhyloNetworks following the discussion by Hibbins and Hahn (2022).

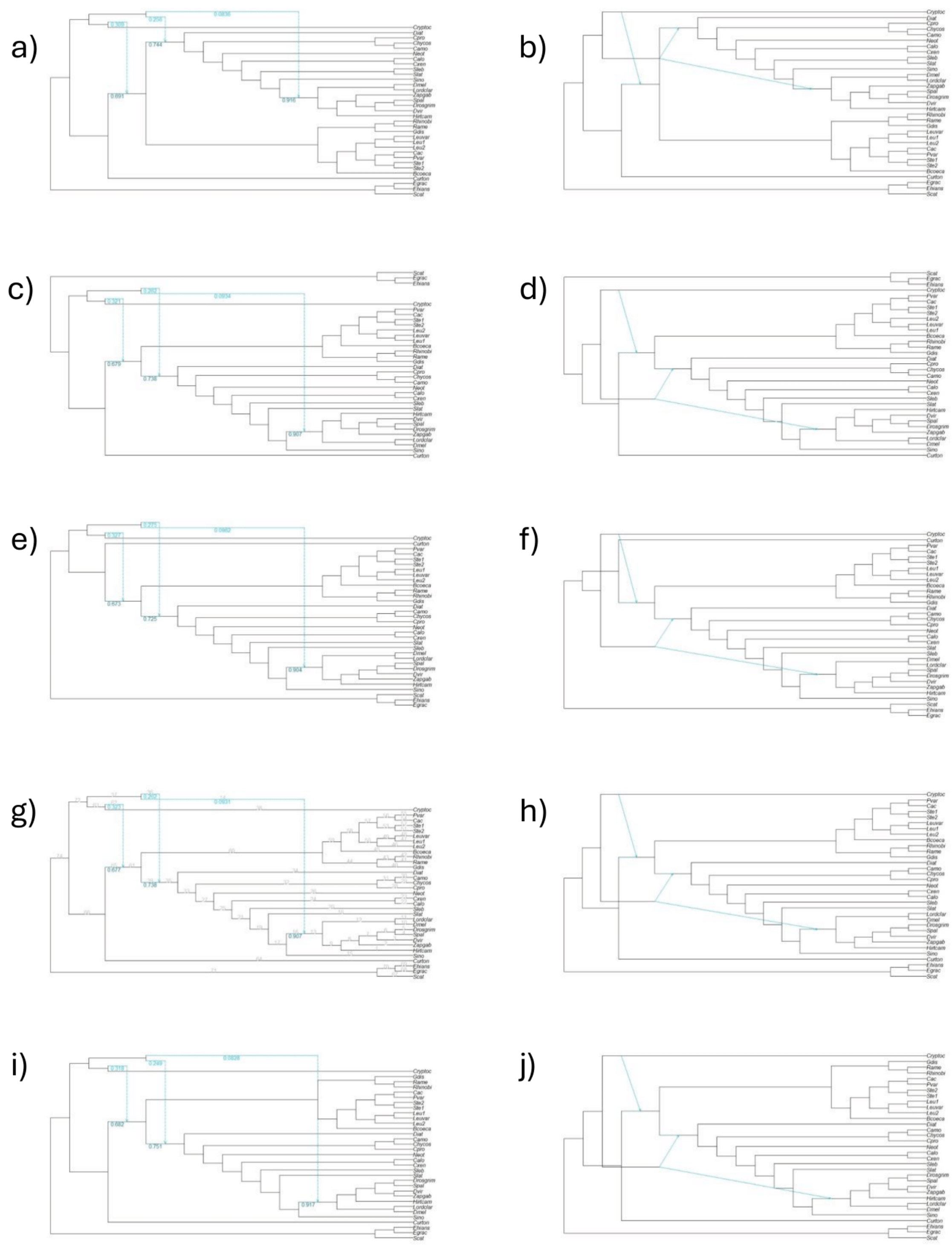

Fig. 2. Phylogenetic networks obtained from MPL analyses on PhyloNet allowing for 3 reticulation events. Each line presents two visualizations of a same phylogenetic network: on the left column, displaying edges as singles lines and showing inheritance values; on the right, displaying hybrid edges as diagonal lines. The five networks differ only on the position

of rogue taxa (*e.g.*, *Scaptodrosophila lebanonensis*) but not on the position of the reticulation events.

The five networks allowing three reticulations indicated introgression events between the following lineages:

i) The ancestor of *Cryptochetum* (or a related lineage) and the ancestor of Drosophilidae;

ii) A "ghost" lineage, sister to Cryptochetidae, and the ancestor of the subfamily Drosophilinae;

iii) A "ghost" lineage, sister to Cryptochetidae, and the ancestor of the *Drosophila* genus.

The events suggested by Phylonet would not explain the main inconsistencies of our phylogenetic hypotheses (*e.g.*, the position of *Scaptodrosophila* species) but highlight that horizontal gene flow may have played a role in the evolution of Drosophilidae.

References:

Wen D, Yu Y, Zhu J, Nakhleh L. 2018. Inferring Phylogenetic Networks Using PhyloNet. Syst. Biol 67:735–740.

Hibbins MS, Hahn MW. 2022. Phylogenomic approaches to detecting and characterizing introgression. Genetics 220.

Solís-Lemus C, Bastide P, Ané C. 2017. PhyloNetworks: A package for phylogenetic networks. Mol. Biol. Evol. 34:3292–3298.

Wen D, Yu Y, Zhu J, Nakhleh L. 2018. Inferring Phylogenetic Networks Using PhyloNet. Syst. Biol 67:735–740.

Supplementary File 3: On the choice of priors for calibration in divergence time estimates

Here we explore two questions: 1) How did the prior choice impact our divergence time estimates; and 2) How did our methods and results differ from Suvorov et al.'s (2021) ones. To address these questions, we conducted three analyses using the same dataset and parameters, changing only the distribution of priors used for calibration. Each analysis was conducted twice to evaluate if the MCMCs converged (which they did, as shown in Fig. 1).

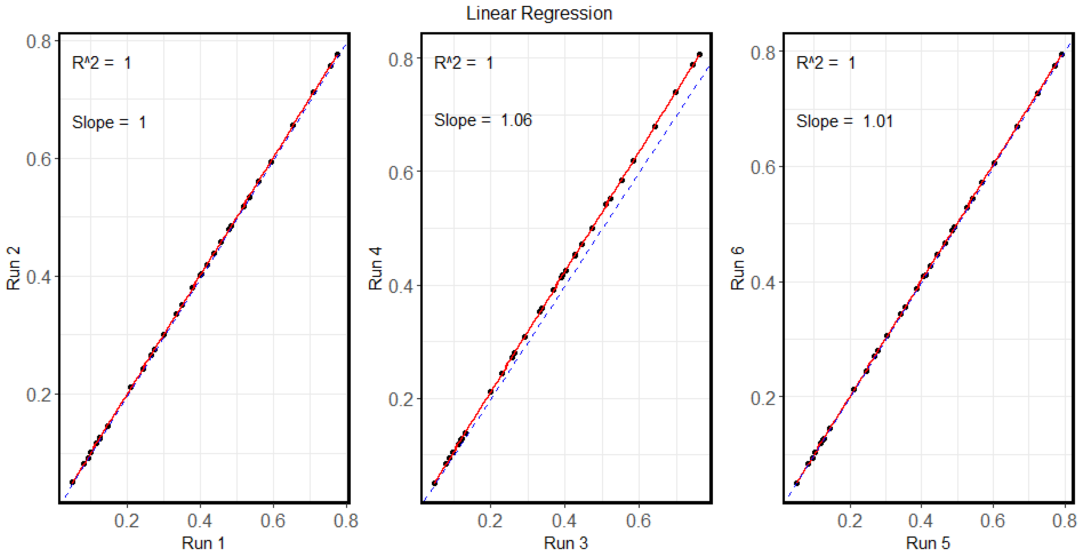

Fig. 1. Convergence of the two independent MCMC runs of each analysis. Blue dashed lines represent slope=1. Red solid lines indicate the regression line through the origin between the time estimates derived from the two independent runs.

The priors' distributions used in each analysis were as follows (Fig. 2):

- Runs 1 and 2: priors with skew-normal distribution
- Runs 3 and 4: priors with uniform distribution
- Runs 5 and 6: priors with normal distribution

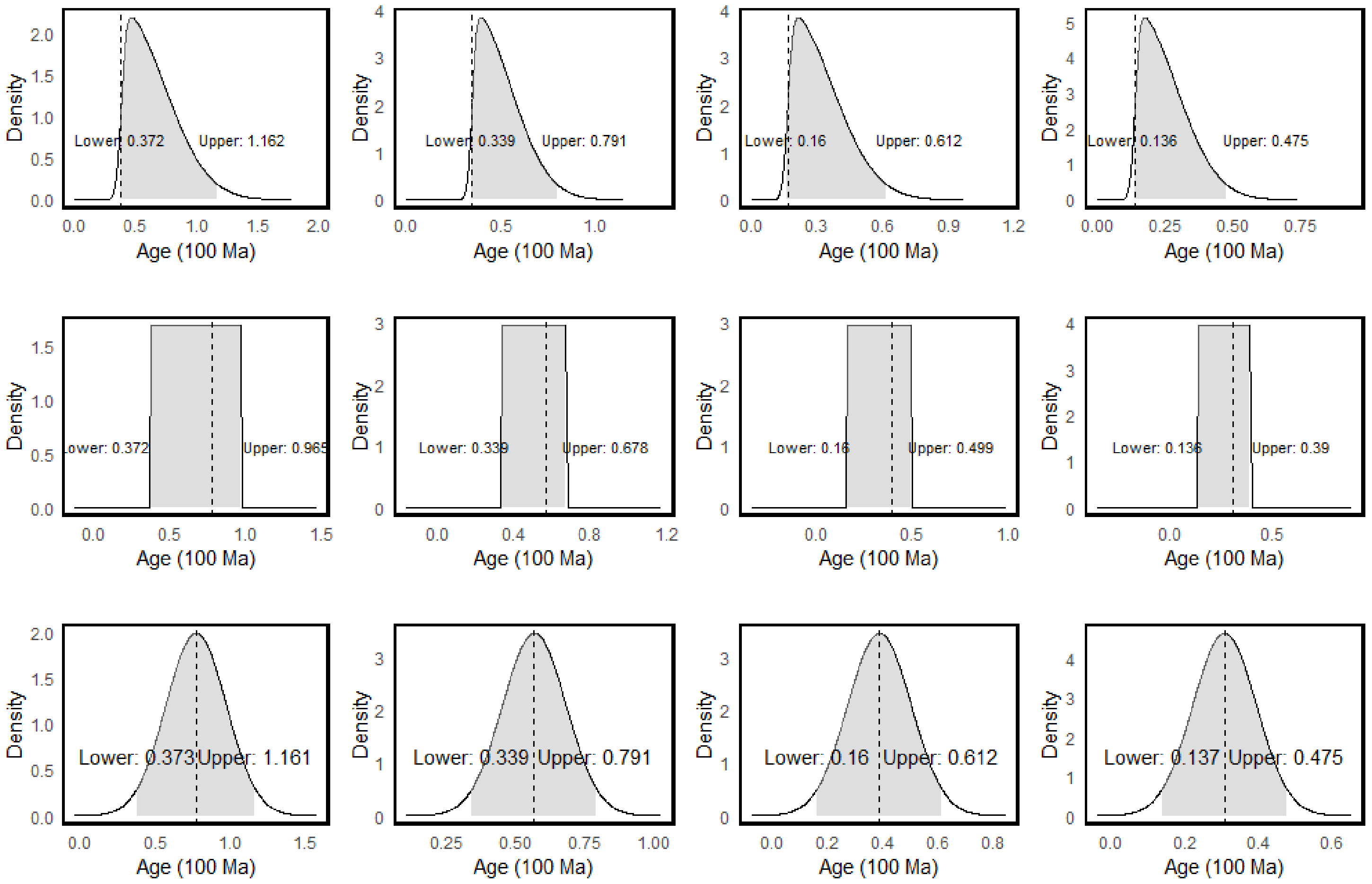

Fig. 2. Priors used in analyses 1 and 2 (first row, skew-normal distribution); 3 and 4 (second row, uniform distribution); and 5 and 6 (third row, normal distribution). Each column shows equivalent priors, which were used to calibrate, from left to right: 1) the Ephydroidea MRCA; 2) the Drosophilidae MRCA; 3) the MRCA of the crown-group including *Neotanygastrella*, *Chymomyza, Colocasiomyia*, *Scaptodrosophila*, *Scaptomyza* and *Drosophila*; 4) the divergence between *Scaptomyza palmae* and *Drosophila virilis*.

As explained in the main text, we used fossils to determine the upper and lower bounds of the 95% interval of the priors with skew-normal distributions (runs 1 and 2). Then, we calculated uniform (runs 3 and 4) and normal (runs 5 and 6) priors so the lower and upper bounds of their 95% intervals would be the same as the ones used in the skew-normal priors. We encountered an issue with the convergence of the MCMC chains when using the uniform priors (analyses 3 and 4). We observed that, when using a wide interval for the prior, the MCMC chains remained stuck in the same region of the parameter space. To address this, we reduced the width of uniform priors' upper bounds by 25%. The reduction effectively allowed the MCMC algorithm to achieve convergence. That's why the upper bounds of uniform priors in Fig. 2 differ from the ones of skew-normal and normal priors.

How did priors influence node age estimates?

After estimating time trees in MCMCTree, we conducted further analyses to evaluate how prior choice influenced the node age estimates. The results show that the ages of older nodes vary more between analyses than those of younger ones (Fig. 3). This pattern is expected since higher values of an estimated parameter will have greater absolute error measures. However, the relative difference should be the same. To test if this was the case, we calculated for each node the coefficient of variation of the mean age, which measures the ratio of the standard deviation to the mean. This coefficient showed that the node age differences between deeper nodes are comparable with that from shallower nodes (Fig. 4).

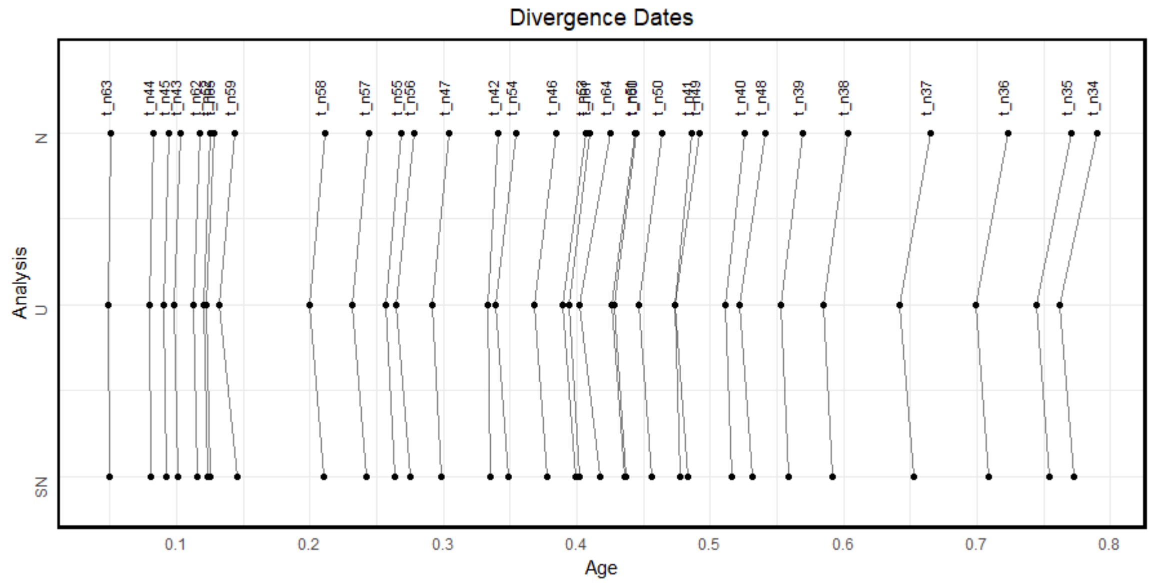

Fig. 3. Mean node ages estimated in the three analyses. As runs converged, each horizontal line represents one run from each analysis (SN: skew-normal priors; U: uniform priors; N: normal priors).

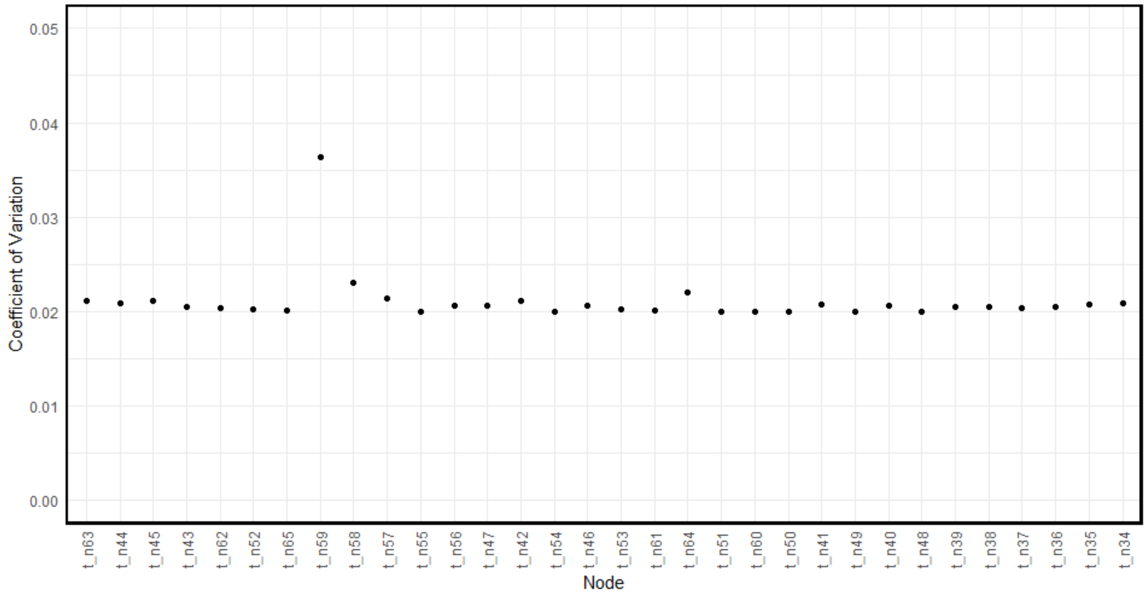

Fig. 4. Coefficient of variations of the mean ages. Nodes are sorted in the x-axis from the younger (t_n63, left) do the older (t_n34, right).

After analyzing how the prior choice affected the estimated mean ages, we investigated how it affected the credibility intervals — in this case, the 95% highest posterior density (HPD) intervals. For this purpose, we computed, for each node, the ratio between the 95% HPD interval and the mean estimated age. Uniform priors provided wider HPD intervals when compared to normal and skew-normal priors (Fig. 5). This is an expected pattern, since uniform distributions represents less informative priors that normal and skew-normal distributions.

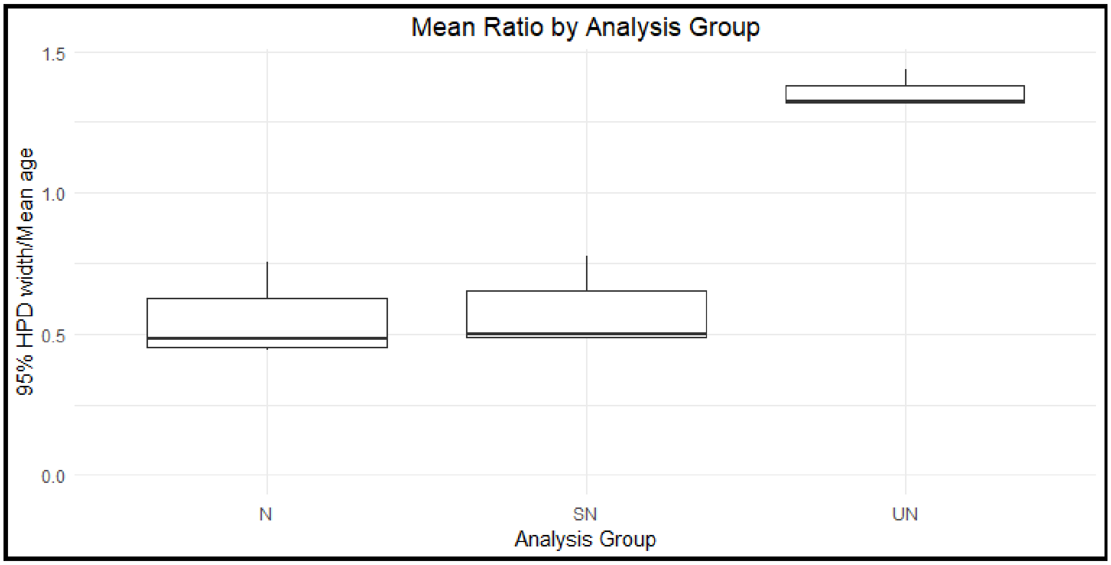

Fig. 5. The use of uniform priors in MCMCTree results provided wider 95% HPD intervals. Each boxplot are the ratios between the 95% HPD width and the mean node ages.

In summary, we concluded that the choice of prior distributions did not significantly alter the mean age estimates. Yet, it did affect the width of the 95% HPD intervals: while skew-normal and normal priors produced very similar results, the less-informative uniform priors produced wider intervals. Overall, our results suggest that the prior distribution had a small effect on the conclusions. This is particularly true for skew-normal vs normal priors.

How do our results compare to Suvorov et al.'s (2021)?

The following table compares some of their estimates with ours.

Table 1. Comparison between our estimates and Suvorov et al.'s (2021).

Split	*Scaptomyza – Drosophila virilis*				*Sophophora-Drosophila*				Drosophilidae MRCA			
	Mean	95% HPD - lower	95% HPD - upper	95% HPD width	Mean	95% HPD - lower	95% HPD - upper	95% HPD width	Mean	95% HPD - lower	95% HPD - upper	95% HPD width
SUVOROV A	30.1	27.7	32.7	5.0	46.8	43.8	49.8	6.0	63.2	59.8	65.7	5.9
SUVOROV B	28.5	25.4	31.4	6.0	45.2	41.1	49.2	8.1	62.2	57.0	65.2	8.3

**SUVOROV C**	31.2	28.2	34.1	5.9	48.0	43.4	51.4	8.0	63.4	59.6	66.1	6.5
**SUVOROV D**	28.5	23.0	32.8	9.8	45.0	36.7	49.3	12.6	61.4	51.4	65.1	13.7
**SUVOROV "RUSSO"**	35.4	29.6	48.1	18.5	55.1	46.3	75.2	28.9	80.4	65.9	109.0	43.1
**Run1 (skew-normal)**	21.0	16.0	26.3	10.2	34.9	26.4	43.4	17.0	65.2	49.3	81.3	31.9
**Run3 (normal)**	19.9	7.8	33.4	25.6	33.9	13.5	56.6	43.1	64.2	25.5	107.1	81.6
**Run5 (uniform)**	21.1	15.6	26.7	11.1	35.4	27.2	43.9	16.7	66.5	51.6	81.3	29.7

Comparing the two studies, both found similar mean ages for the older nodes (e.g., the Drosophilidae MRCA), while ours obtained more recent mean ages for the younger nodes (*e.g.*, the *Scaptomyza-Drosophila virilis*). Also, we obtained wider 95% HPD intervals, suggesting a greater uncertainty in our estimates. These discrepancies could have two causes: taxon sampling and prior choice.

In terms of taxon sampling, the two studies differed in focus. While Suvorov et al. (2021) extensively sampled *Drosophila* species, we focused on the relationships outside of *Drosophila*, sampling more comprehensively from other Drosophilidae genera in both the Drosophilinae and Steganinae subfamilies.

As for the choice of priors, we opted for more conservative ones. Most of Suvorov et al.'s (2021) calibration schemes used uniform priors with mean widths that were shorter than ours: 56.5 Ma vs 28 Ma (in runs 3 and 4, which used uniform priors; as explained above, the other priors were still 25% wider). The rationale for our decisions regarding prior boundaries is expressed in the section "Fossil information and node calibration" of the main paper. Suvorov et al. (2021) prior widths were also based on fossils, but their decisions are less conservative than ours. For instance, to calibrate the Drosophilidae MRCA, they used two fossils: *Electrophortica succini* (43 Ma), to set a lower bound, and *Phytomyzites sp.* (64 Ma), to set an upper bound. However, *Phytomyzites sp.*, despite belonging to Schizophora, is not part of the Ephydroidea superfamily. Hence, its existence in 64 Ma does not preclude Drosophilidae to have originated earlier. We took a more conservative approach: we also used *Electrophortica succini* (37.2-33.9) to set a lower bound, but we set the upper bound to 79.1 Ma based on the ages of other Schizophora and Cyclorrapha fossils. Furthermore, note that the dates used for the *Electrophortica succini* also varied between the two studies: Suvorov used it to set the lower bound at 43 Ma, while we used to set it at 33.9 Ma. Our data was retrieved from the Paleobiology Database, accessed on 21 June 2020 via the Fossilworks gateway (Alroy 2020).

Finally, the two studies used the same fossils to calibrate different nodes of the tree, and this could explain the differences obtained for the younger nodes. For the separation between *Scaptomyza* and *Drosophila virilis*, we used a prior with a 95% interval between 13.65 and 47.5 Ma, basing the lower bound on the fossil *Scaptomyza americana* (13.65-20.43 Ma). Suvorov et al (2021) used a similar calibration for the clade "*Scaptomyza* stem" (15-43 Ma), which is younger than the *Scaptomyza-D. virilis* separation. So, our calibration point, besides being older, was calibrated similarly, and thus we recovered younger ages for the more recent splits.

In summary, by choosing less conservative priors Suvorov et al. (2021) obtained narrower 95% HPD intervals. However, their upper bounds, as ours, are arbitrary, and the short range of their results may be masking the uncertainty about the age of some nodes. Also, MCMCTree—used in both studies—does not accounts for gene tree heterogeneity and thus may underestimate divergence times and provide shorter and unreliable confidence intervals (Tiley et al. 2023). Thus, we believe that our approach, while yielding wider confidence intervals, provides a more realistic representation of the uncertainties inherent in such analyses. Despite the differences, overall, both Suvorov et al.'s (2021) and our results are compatible with the fossils and evolutive scenarios previously proposed for Drosophilidae (*e.g.*, Russo et al. 1995; Tamura et al. 2004; Gao et al. 2011; Obbard et al. 2012; Russo et al. 2013; Katoh et al. 2017).

References

Alroy J. 2020. Fossilworks: gateway to the paleobiology database. Fossilworks [Internet]. Available from: http://fossilworks.org

Gao JJ, Hu YG, Toda MJ, Katoh T, Tamura K. 2011. Phylogenetic relationships between Sophophora and Lordiphosa, with proposition of a hypothesis on the vicariant divergences of tropical lineages between the Old and New Worlds in the family Drosophilidae. Mol. Phylogenet. Evol. [Internet] 60:98–107. Available from: http://dx.doi.org/10.1016/j.ympev.2011.04.012

Katoh T, Izumitani HF, Yamashita S, Watada M. 2017. Multiple origins of Hawaiian drosophilids: Phylogeography of Scaptomyza Hardy (Diptera : Drosophilidae). Entomol. Sci. 20:33–44.

Obbard DJ, Maclennan J, Kim K, Rambaut A, Grady PMO, Jiggins FM. 2012. Estimating Divergence Dates and Substitution Rates in the Drosophila Phylogeny. Mol. Biol. Evol. 29:3459–3473.

Russo CA, Takezaki N, Nei M. 1995. Molecular phylogeny and divergence times of drosophilid species. Mol Biol Evol 12:391–404.

Russo CAM, Mello B, Frazão A, Voloch CM. 2013. Phylogenetic analysis and a time tree for a large drosophilid data set (Diptera : Drosophilidae). Zool. J. Linn. Soc. 169:765–775.

Tamura K, Subramanian S, Kumar S. 2004. Temporal Patterns of Fruit Fly (Drosophila) Evolution Revealed by Mutation Clocks. Mol. Biol. Evol. 21:36–44.

Supplementary file 4: Source of sequenced samples

Rhinoleucophenga americana. We used 15 adult females from a lineage obtained from the San Diego Drosophila Species Stock Center (USA), lineage 70000-3000.00, and bred in the Carvalho laboratory in Rio de Janeiro, Brazil. The founders of the colony were collected by Maxi Richmond on the coast of San Diego, CA, USA (32°43' N; 117°15' W), emerging from a rotting prickly pear fruit (*Opuntia sp.*).

Rhinoleucophenga cf. bivisualis. As explained in Dias et al. (2020), we sequenced the genome from a single male specimen collected in the Brazilian Cerrado by Rosana Tidon and Juliana Miranda. These specimens were identified as *Rhinoleucophenga bivisualis*, but we considered the possibility that they may belong to *Rhinoleucophenga punctulata*, as noted by Vilela and Bachli (2009). The reasons are: first, the two species have spotted thorax and similar external morphology, and differ mainly in the unusual, divided type of eye of the first, apparently only seen when the flies are alive. Second, specimens of *Rhinoleucophenga punctulata* will run to *Rhinoleucophenga bivisualis* (cited as *Gitona*) in two commonly used keys (Wheeler 1949; Wheeler 1952) that include the latter but not the former species. Finally, *Rhinoleucophenga punctulata* is most probably endemic to the South American Chaco and Cerrado biomes, while *Rhinoleucophenga bivisualis* is apparently endemic to the Nearctic Region. Thus, since our specimen was collected in the Brazilian Cerrado and no further information about its taxonomy is available, we believe it is prudent to account for the possibility that it may belong to *Rhinoleucophenga punctulata.*

Gitona distigma. We used one adult male collected and identified by Gerard Bächli in Leuk, Switzerland. It emerged from flower heads (capitula) of *Sonchus arvensis* (Asteraceae) in 09.IX.2019.

***Leucophenga sp.* 1.** We used nine thoraxes of adult females collected and identified by Marco Gottschalk in Ribeirão Preto, SP, Brazil. The specimens were collected on the USP campus, in front of the FAMED building, on 09/VII/2017.

***Leucophenga sp.* 2.** We used two adult females collected and identified by Marco Gottschalk in Ribeirão Preto, SP, Brazil. The specimens were collected on the USP campus, in front of the 07 FFCLRP building.

***Stegana sp.* 1.** We used the thorax of a single adult female collected in December 2008 by C. Vilela and Carvalho lab members in Itatiaia, RJ, Brazil, and identified by C. Vilela.

***Stegana sp.* 2.** We used the thorax of a single adult female collected in December 2008 by C. Vilela and Carvalho lab members in Itatiaia, RJ, Brazil, and identified by C. Vilela.

Scaptomyza palmae. We used 30 adult males from the National Drosophila Stock Center (Cornell U., USA), lineage 33000-2681.01.

Scaptodrosophila inornata. We used the thorax of a single adult female collected by Michael Polak and William T. Starmer in Australia around 2007 with resources from the International Programs at the National Science Foundation (NSF, INT-9820674).

***Scaptodrosophila latifasciaeformis*.** We used the thoraxes from ten adult females from San Diego Drosophila Species Stock Center lineage 11030-0061.01.

***Colocasiomyia alocasiae*.** We used 20 adult females collected and identified by T. Miyake and M. Yafuso in Japan in 2019.

Neotanygastrella sp. We used a single adult male collected in December 2010 by A.B. Carvalho in Ubatumirim, SP, Brazil, and identified by S. Vaz.

Chymomyza procnemis. We used 30 adult females from an isogenic lineage bred in the Carvalho laboratory in Rio de Janeiro, Brazil (CpJ211aa10aa; 9 generations inbreeding).

The lineage was provided by Celeste Berg (UCSD Stock Center *Chymomyza procnemis* line 2000-2640.00, Japan).

Diathoneura tesselata**.** We used 30 adult males collected in 2007 by Glen Collier emerging from *Anaxagorea crassipetala* tepals in Costa Rica (Collier and Armstrong 2009).

Scatella sp. We used 30 virgin adult females from an isogenic lineage bred in the laboratory (ten generations of inbreeding). The founders of the lineage were collected in 20/V/2018 by Fabiana Uno in the Canal de Itaipú (Latitude: 22°57' S, Longitude: 43°02’ W), Niterói, RJ, Brazil.
