# Supplementary material for: Timing and Pattern of Early Diversification in Drosophilidae (Diptera)
Source: Mol Biol Evol. 2025 Oct 23;42(11):msaf269. doi: 10.1093/molbev/msaf269 (PMC12603360; doi:10.1093/molbev/msaf269)
Supplement: msaf269_Supplementary_Data [file msaf269_supplementary_data.zip › MBE25-0386_Supplementary_figures_S1-S23.pdf]

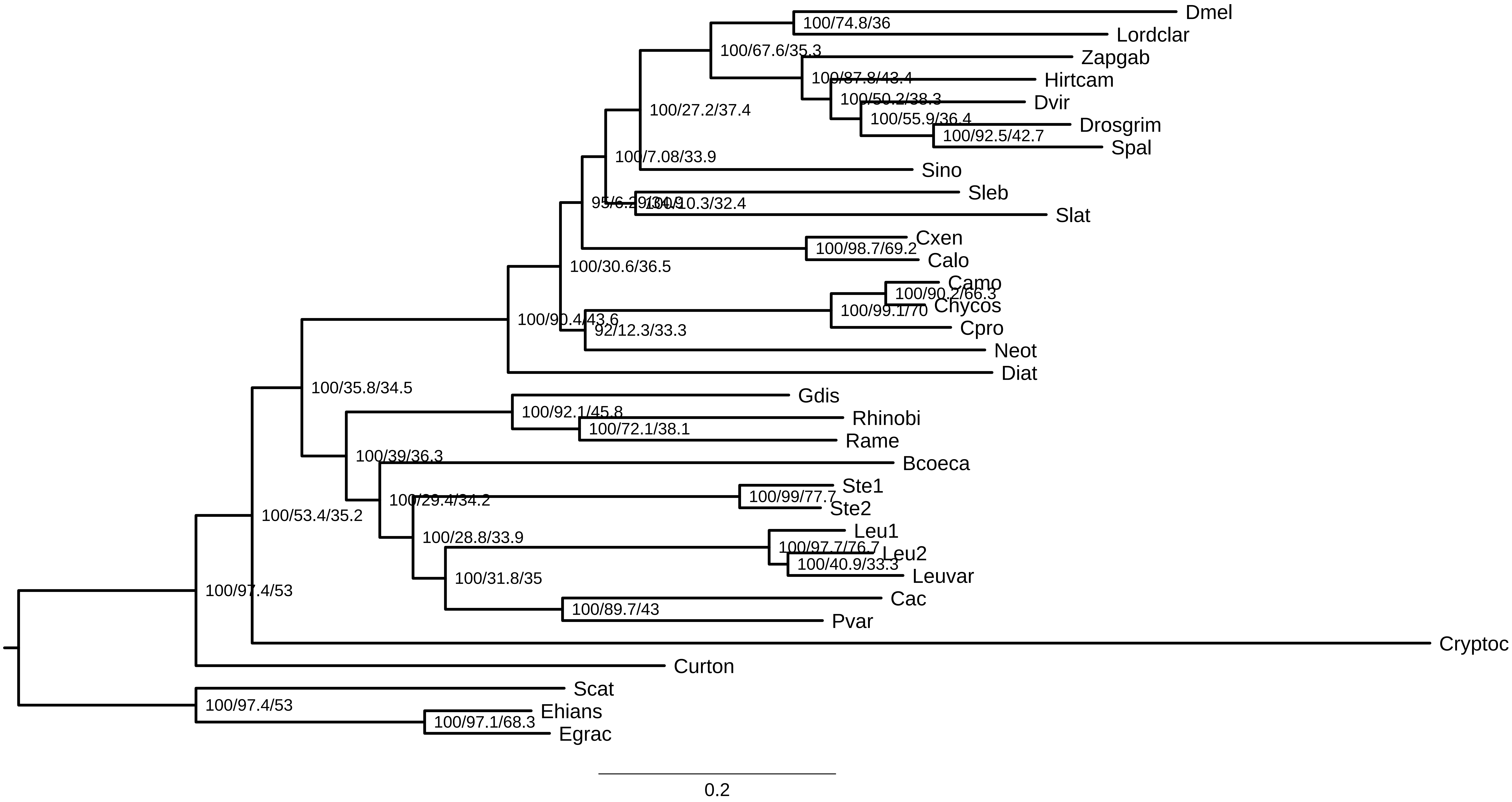
Dmel
100/74.8/36
Lordclar
100/67.6/35.3
Zapgab
100/87.8/43.4
Hirtcam
100/50.2/38.3
Dvir
100/27.2/37.4
100/55.9/36.4
Drosgrim
100/92.5/42.7
Spal
100/7.08/33.9
Sino
Sleb
100/10.3/32.4
Slat
Cxen
100/98.7/69.2
Calo
100/30.6/36.5
Camo
100/90.2/66.3
Chycos
100/99.1/70
100/90.4/43.6
Cpro
92/12.3/33.3
Neot
Diat
100/35.8/34.5
Gdis
100/92.1/45.8
Rhinobi
100/72.1/38.1
Rame
100/39/36.3
Bcoeca
Ste1
100/99/77.7
100/29.4/34.2
Ste2
100/53.4/35.2
Leu1
100/28.8/33.9
100/97.7/76.7
Leu2
100/40.9/33.3
Leuvar
100/31.8/35
100/97.4/53
Cac
100/89.7/43
Pvar
Cryptoc
Curton
Scat
100/97.4/53
Ehians
100/97.1/68.3
Egrac
0.2

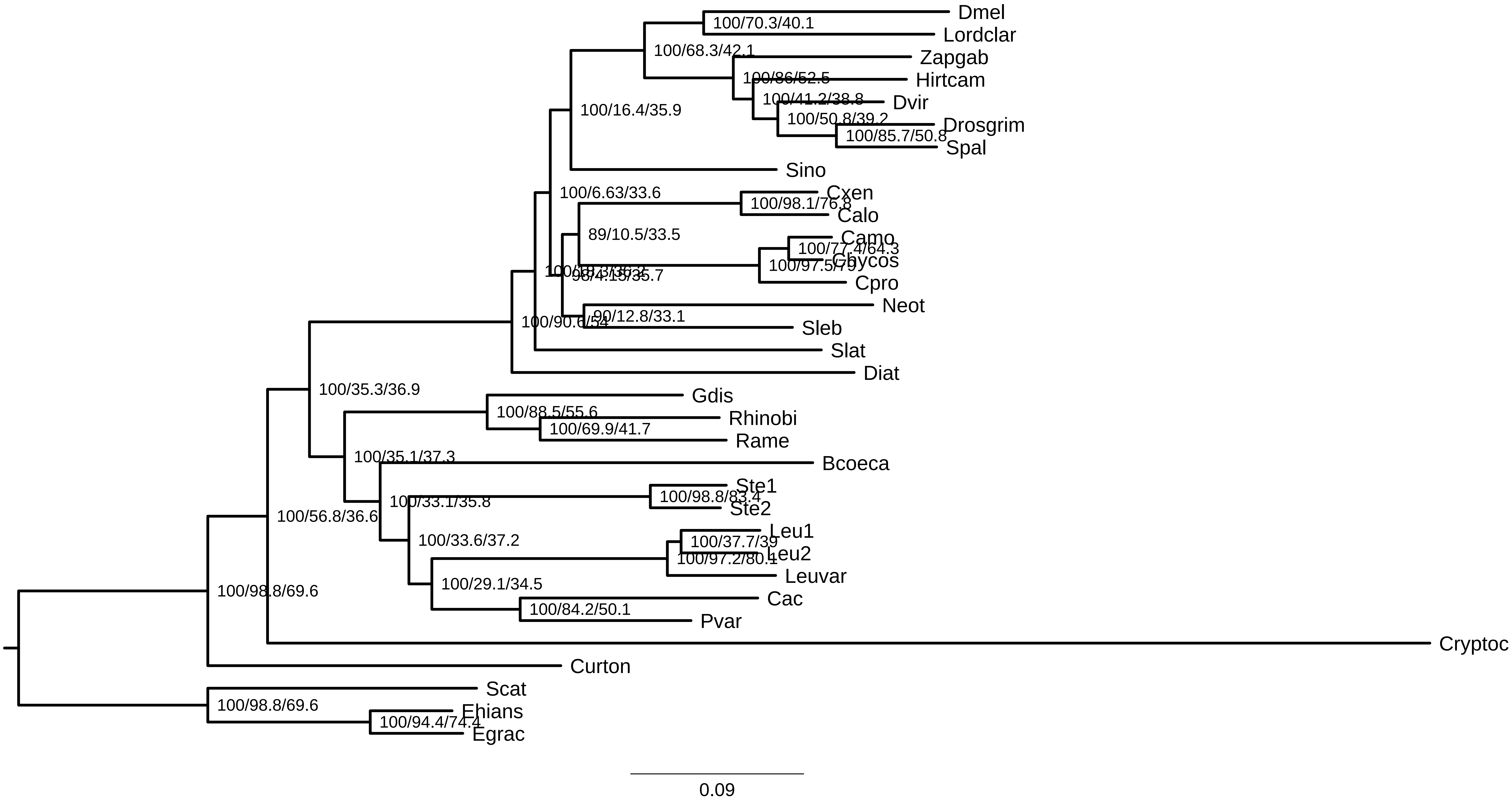
Dmel
Lordclar
Zapgab
Hirtcam
Dvir
Drosgrim
Spal
Sino
Cxen
Calo
Camo
Cpro
Neot
Sleb
Slat
Diat
Gdis
Rhinobi
Rame
Bcoeca
Ste1
Ste2
Leu1
Leu2
Leuvar
Cac
Pvar
Cryptoc
Curton
Scat
Ehians
Egrac
100/70.3/40.1
100/68.3/42.1
100/86/52.5
100/41.2/38.8
100/50.8/39.2
100/85.7/50.8
100/16.4/35.9
100/6.63/33.6
100/98.1/76.8
89/10.5/33.5
100/77.4/64.3
100/97.5/79
98/4.15/35.7
90/12.8/33.1
100/90.6/54
100/35.3/36.9
100/88.5/55.6
100/69.9/41.7
100/35.1/37.3
100/98.8/83.4
100/33.1/35.8
100/56.8/36.6
100/33.6/37.2
100/37.7/39
100/97.2/80.1
100/29.1/34.5
100/84.2/50.1
100/98.8/69.6
100/98.8/69.6
100/94.4/74.4
0.09

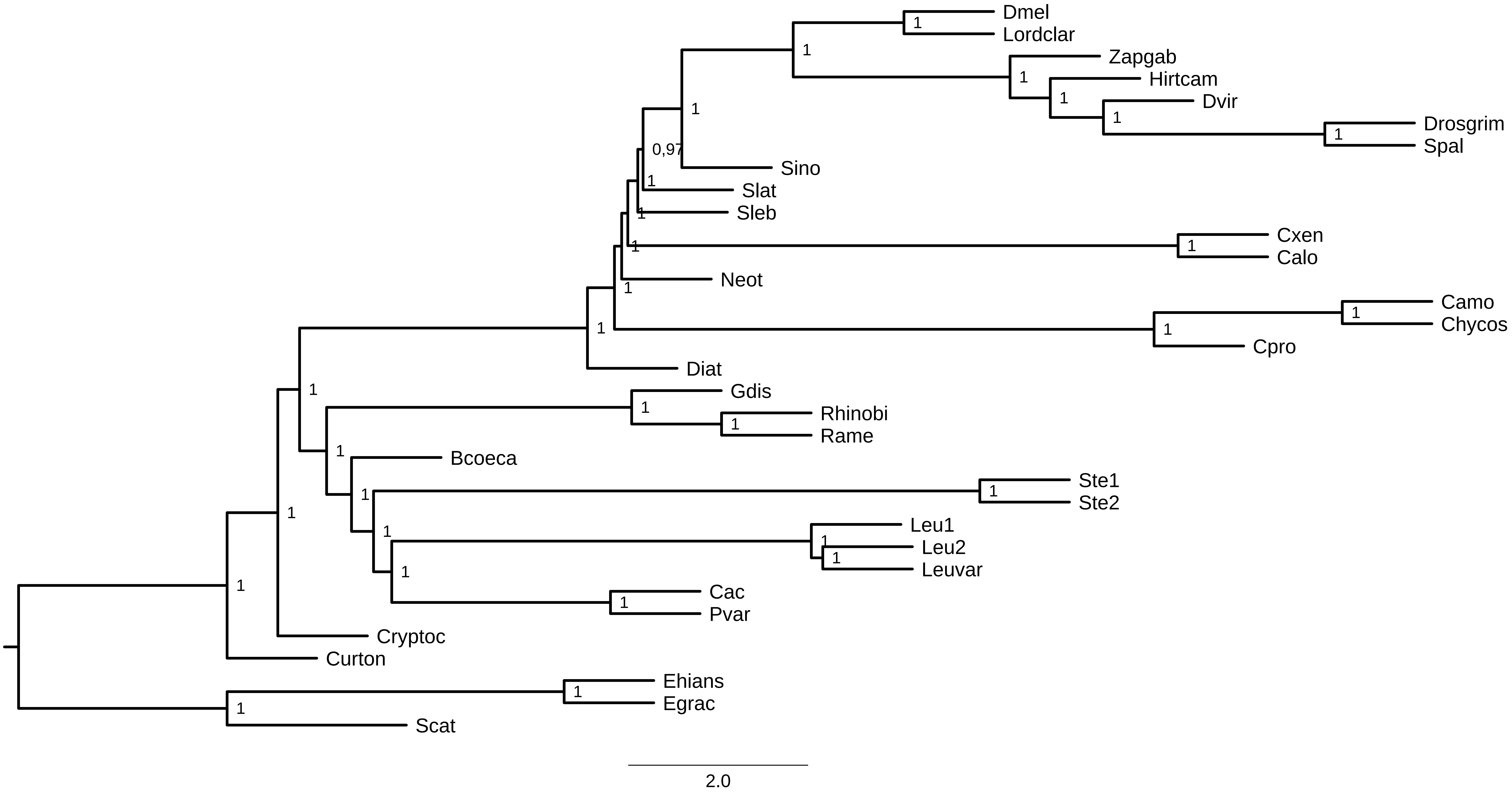

Dmel
Lordclar
Zapgab
Hirtcam
Dvir
Drosgrim
Spal
Sino
Slat
Sleb
Cxen
Calo
Neot
Camo
Chycos
Cpro
Diat
Gdis
Rhinobi
Rame
Bcoeca
Ste1
Ste2
Leu1
Leu2
Leuvar
Cac
Pvar
Cryptoc
Curton
Ehians
Egrac
Scat
0,97
2.0

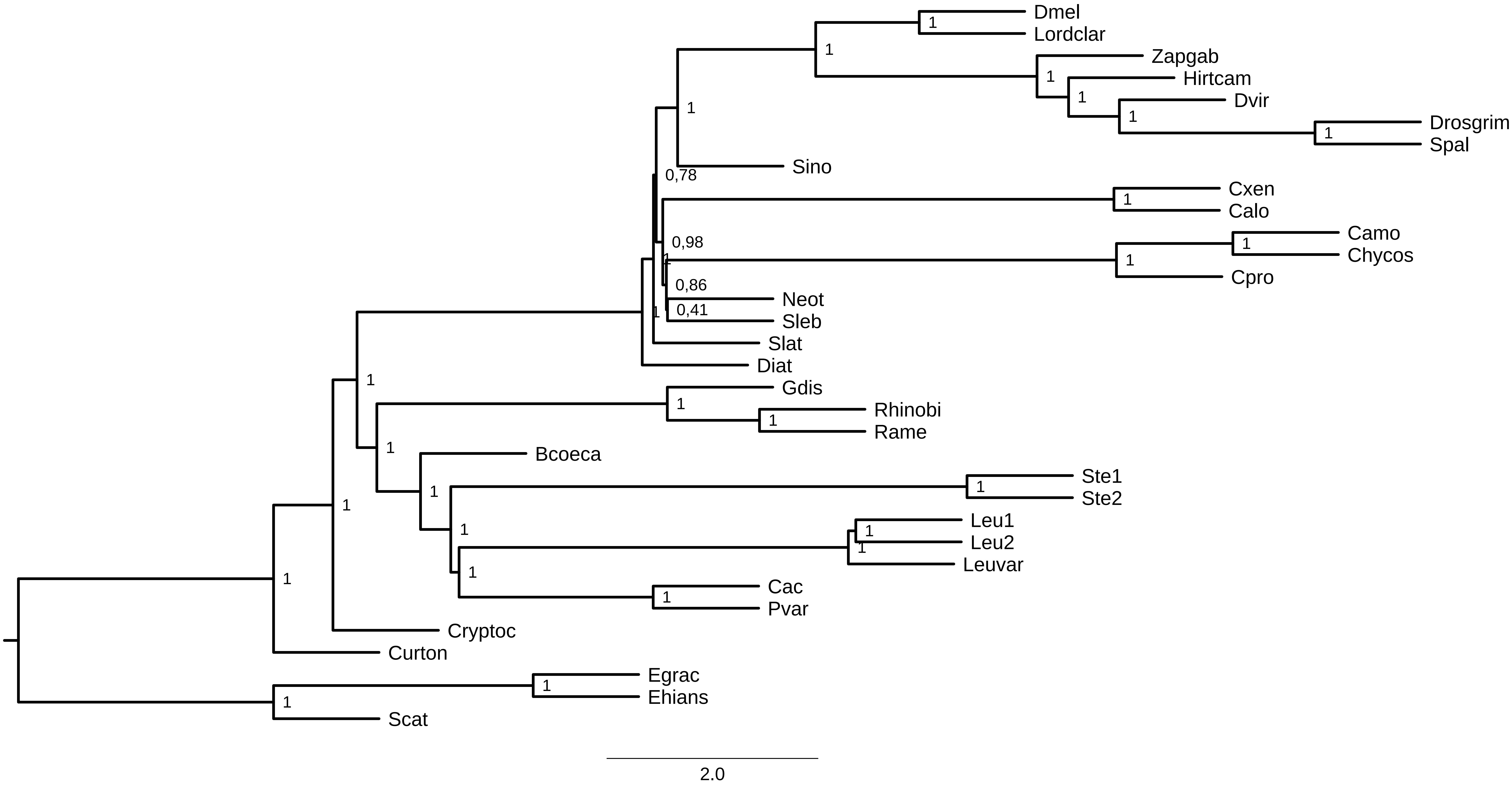
Dmel
Lordclar
Zapgab
Hirtcam
Dvir
Drosgrim
Spal
Sino
Cxen
Calo
Camo
Chycos
Cpro
Neot
Sleb
Slat
Diat
Gdis
Rhinobi
Rame
Bcoeca
Ste1
Ste2
Leu1
Leu2
Leuvar
Cac
Pvar
Cryptoc
Curton
Egrac
Ehians
Scat
0,78
0,98
0,86
0,41
2.0

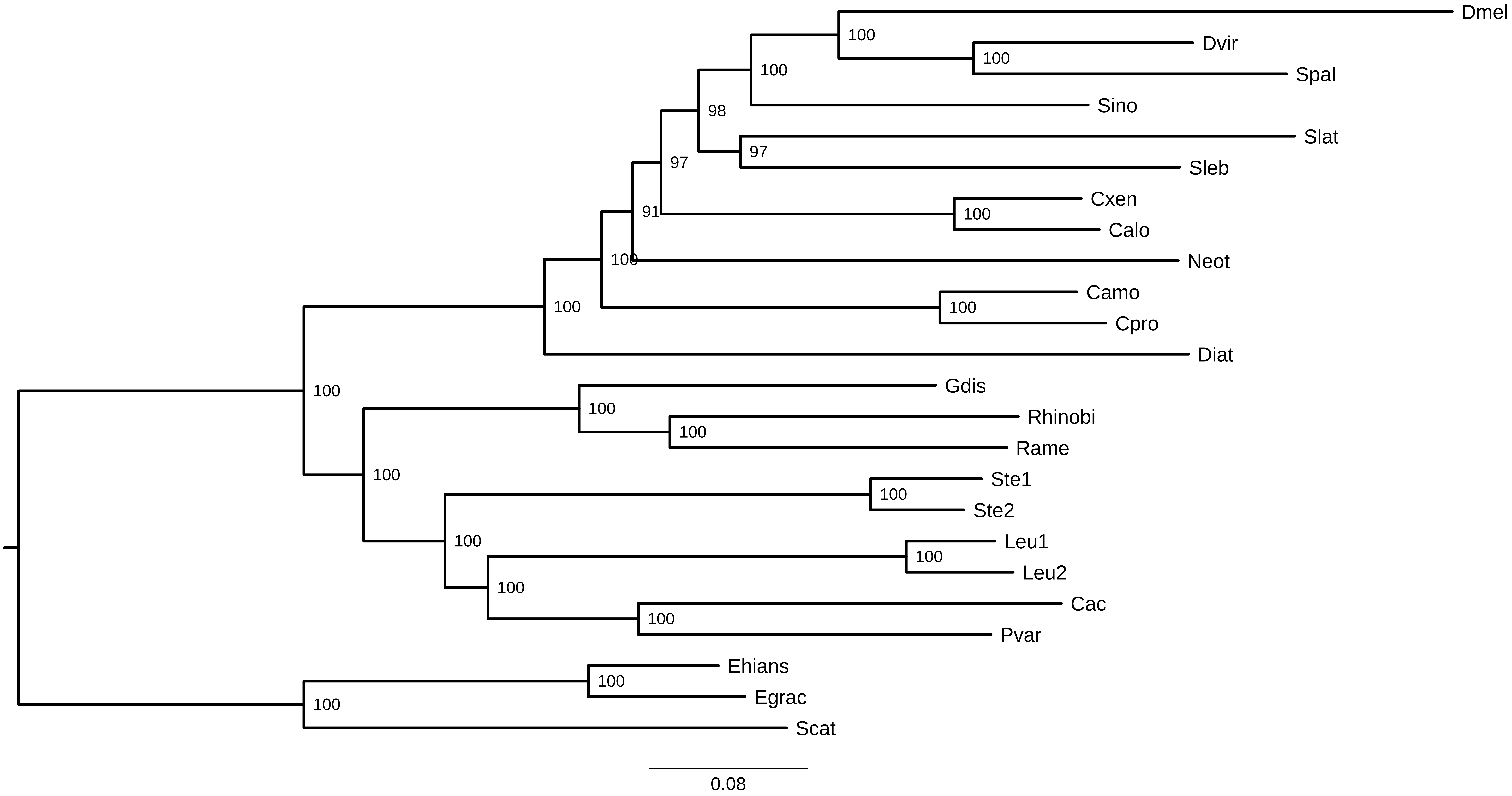
Dmel
Dvir
Spal
Sino
Slat
Sleb
Cxen
Calo
Neot
Camo
Cpro
Diat
Gdis
Rhinobi
Rame
Ste1
Ste2
Leu1
Leu2
Cac
Pvar
Ehians
Egrac
Scat
100
100
100
98
97
97
91
100
100
100
100
100
100
100
100
100
100
100
100
100
100
100
0.08

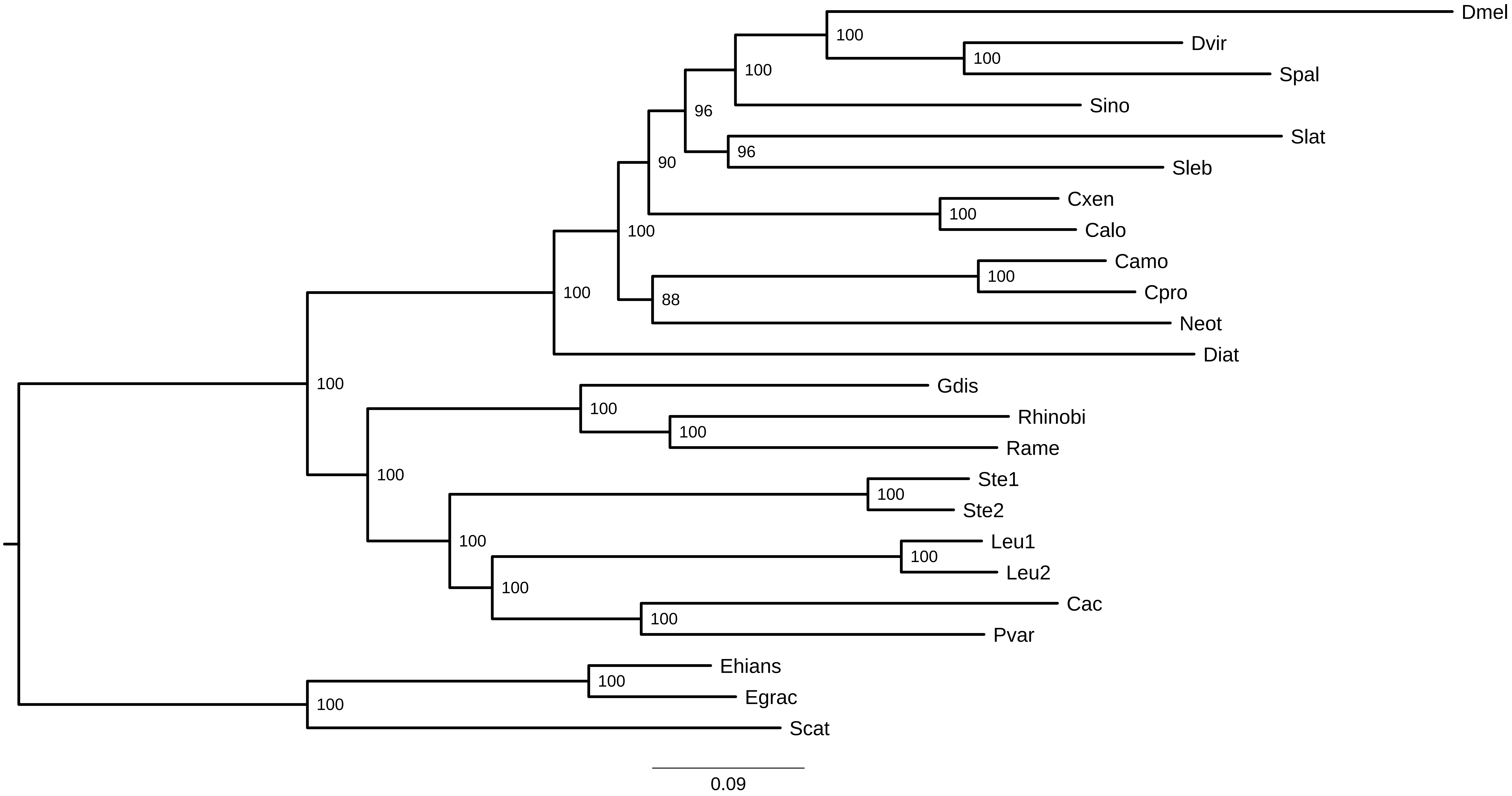
Dmel
Dvir
Spal
Sino
Slat
Sleb
Cxen
Calo
Camo
Cpro
Neot
Diat
Gdis
Rhinobi
Rame
Ste1
Ste2
Leu1
Leu2
Cac
Pvar
Ehians
Egrac
Scat
100
96
90
88
0.09

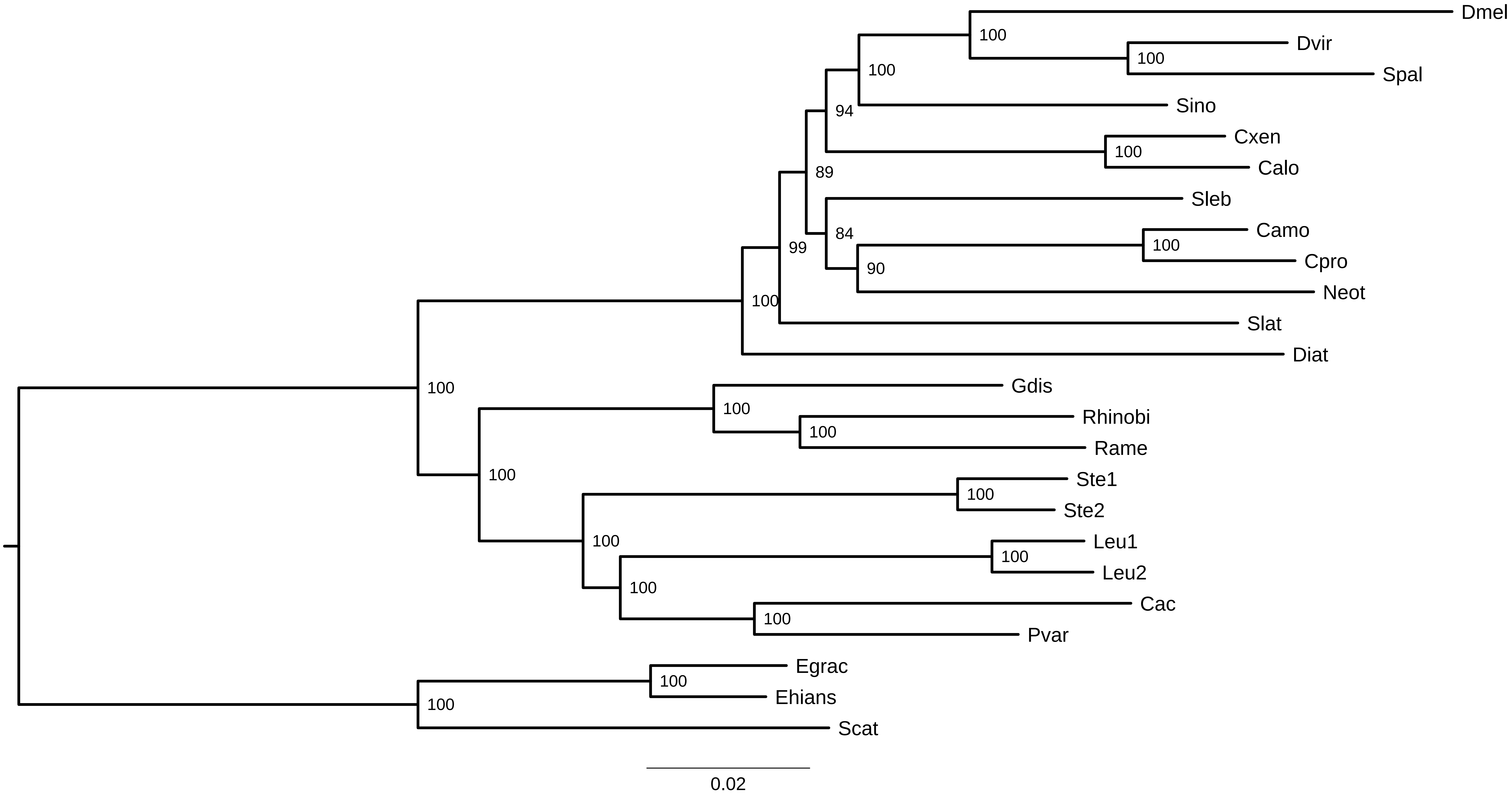

Dmel
Dvir
Spal
Sino
Cxen
Calo
Sleb
Camo
Cpro
Neot
Slat
Diat
Gdis
Rhinobi
Rame
Ste1
Ste2
Leu1
Leu2
Cac
Pvar
Egrac
Ehians
Scat
100
100
100
94
100
89
84
100
90
99
100
100
100
100
100
100
100
100
100
100
100
100
0.02

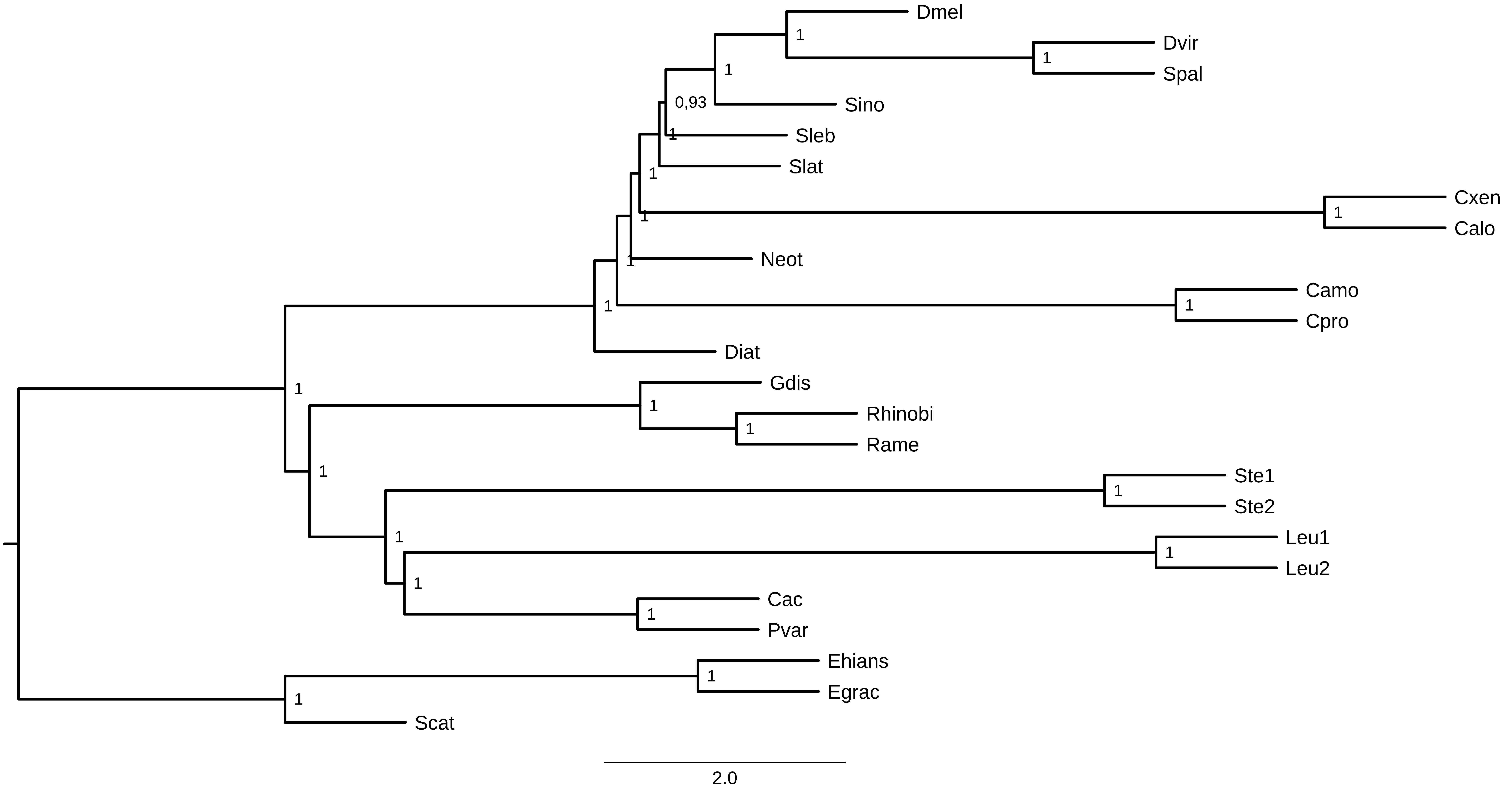
Dmel
Dvir
Spal
Sino
Sleb
Slat
Cxen
Calo
Neot
Camo
Cpro
Diat
Gdis
Rhinobi
Rame
Ste1
Ste2
Leu1
Leu2
Cac
Pvar
Ehians
Egrac
Scat
0,93
2.0

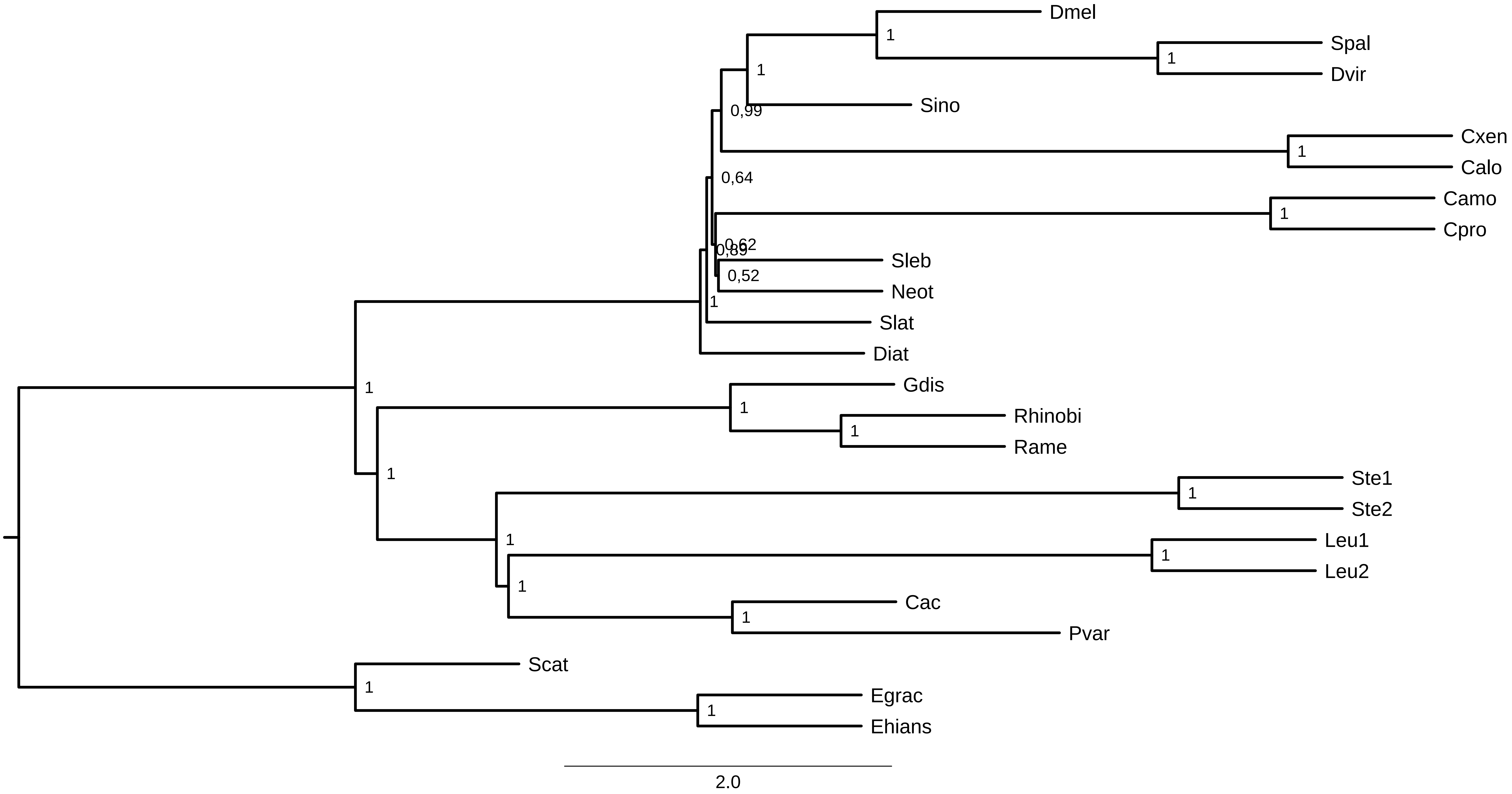
Dmel
1
Spal
1
Dvir
1
Sino
0,99
Cxen
1
Calo
0,64
Camo
1
Cpro
0,62
0,89
Sleb
0,52
Neot
1
Slat
Diat
1
Gdis
1
Rhinobi
1
Rame
1
Ste1
1
Ste2
1
Leu1
1
Leu2
1
Cac
1
Pvar
Scat
1
Egrac
1
Ehians
2.0

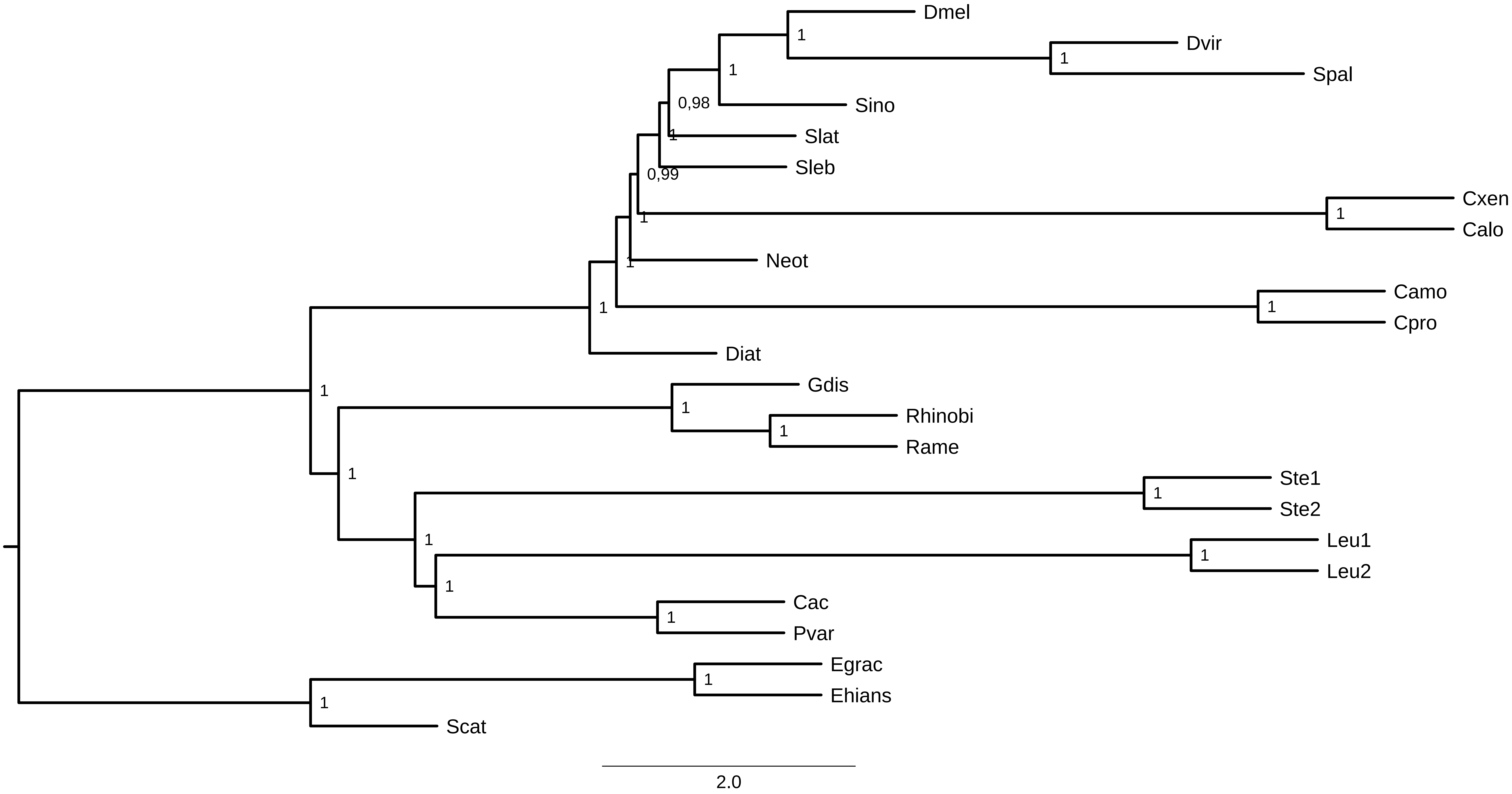
Dmel
Dvir
Spal
Sino
Slat
Sleb
Cxen
Calo
Neot
Camo
Cpro
Diat
Gdis
Rhinobi
Rame
Ste1
Ste2
Leu1
Leu2
Cac
Pvar
Egrac
Ehians
Scat
0,98
0,99
2.0

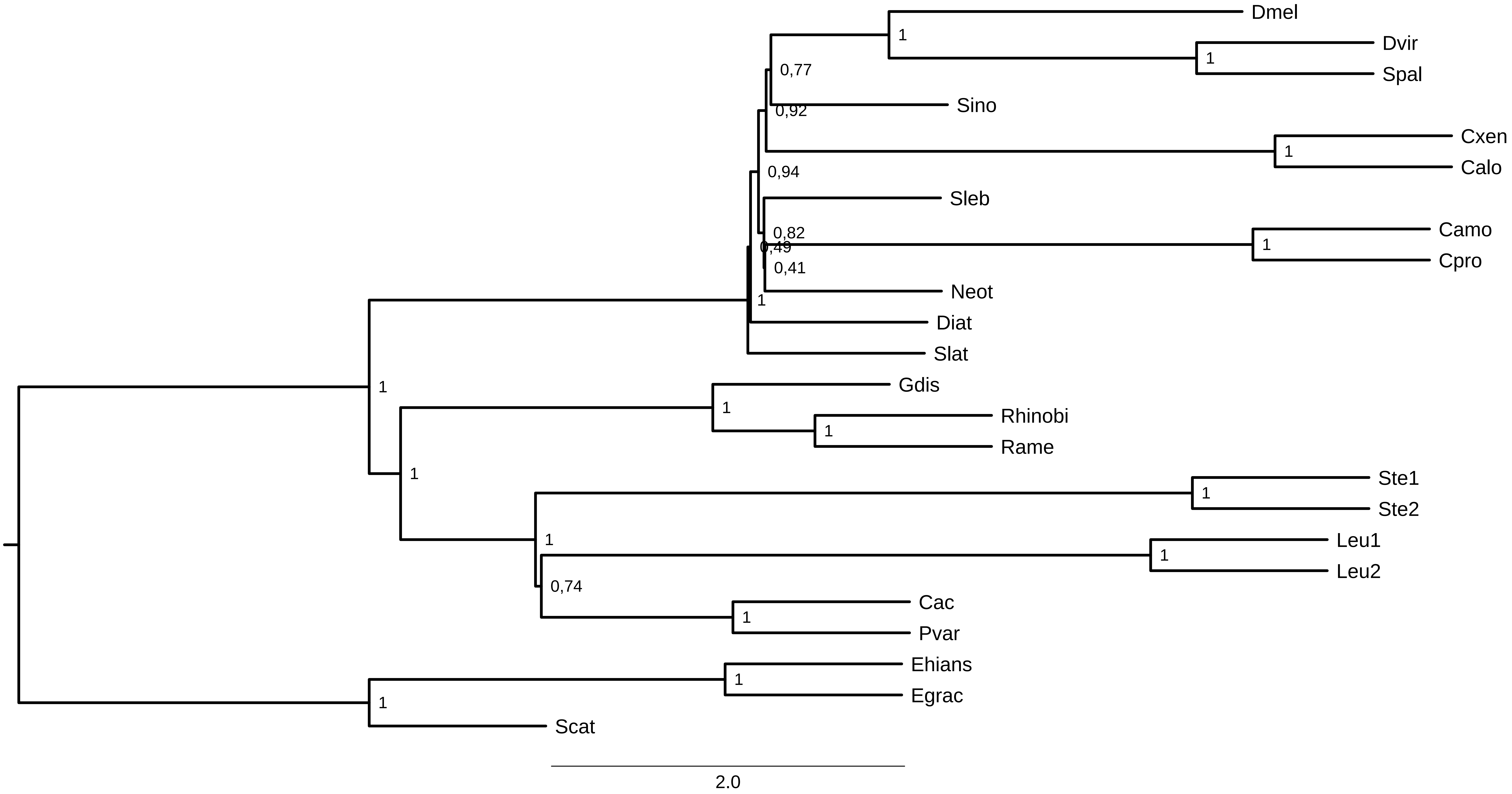

Dmel
1
Dvir
1
Spal
0,77
Sino
0,92
Cxen
1
Calo
0,94
Sleb
0,82
Camo
0,49
1
Cpro
0,41
Neot
1
Diat
Slat
Gdis
1
1
Rhinobi
1
Rame
1
Ste1
1
Ste2
1
Leu1
1
Leu2
0,74
Cac
1
Pvar
Ehians
1
Egrac
1
Scat
2.0

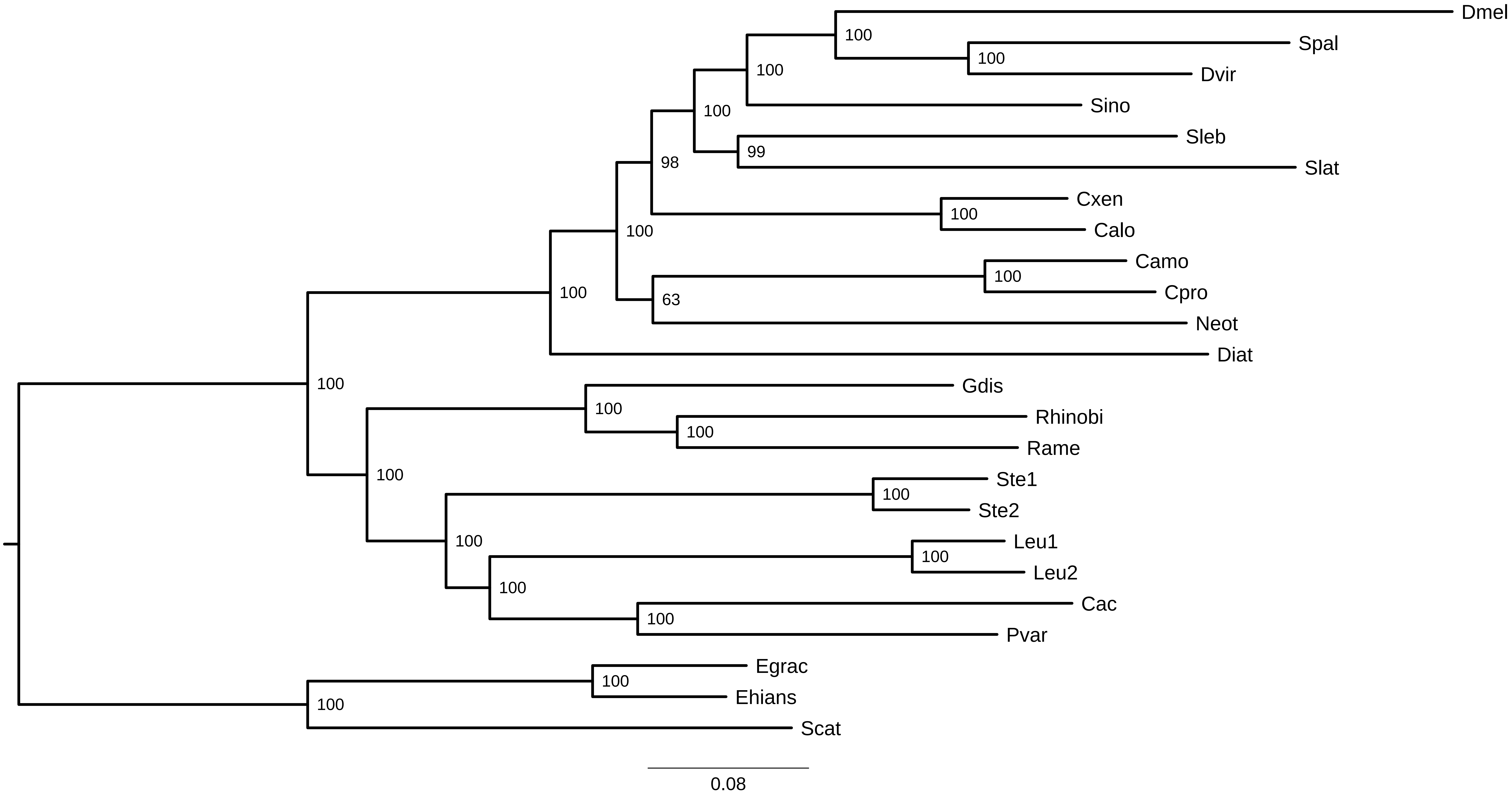
Dmel
Spal
Dvir
Sino
Sleb
Slat
Cxen
Calo
Camo
Cpro
Neot
Diat
Gdis
Rhinobi
Rame
Ste1
Ste2
Leu1
Leu2
Cac
Pvar
Egrac
Ehians
Scat
100
100
100
100
98
99
100
100
100
63
100
100
100
100
100
100
100
100
100
100
100
0.08

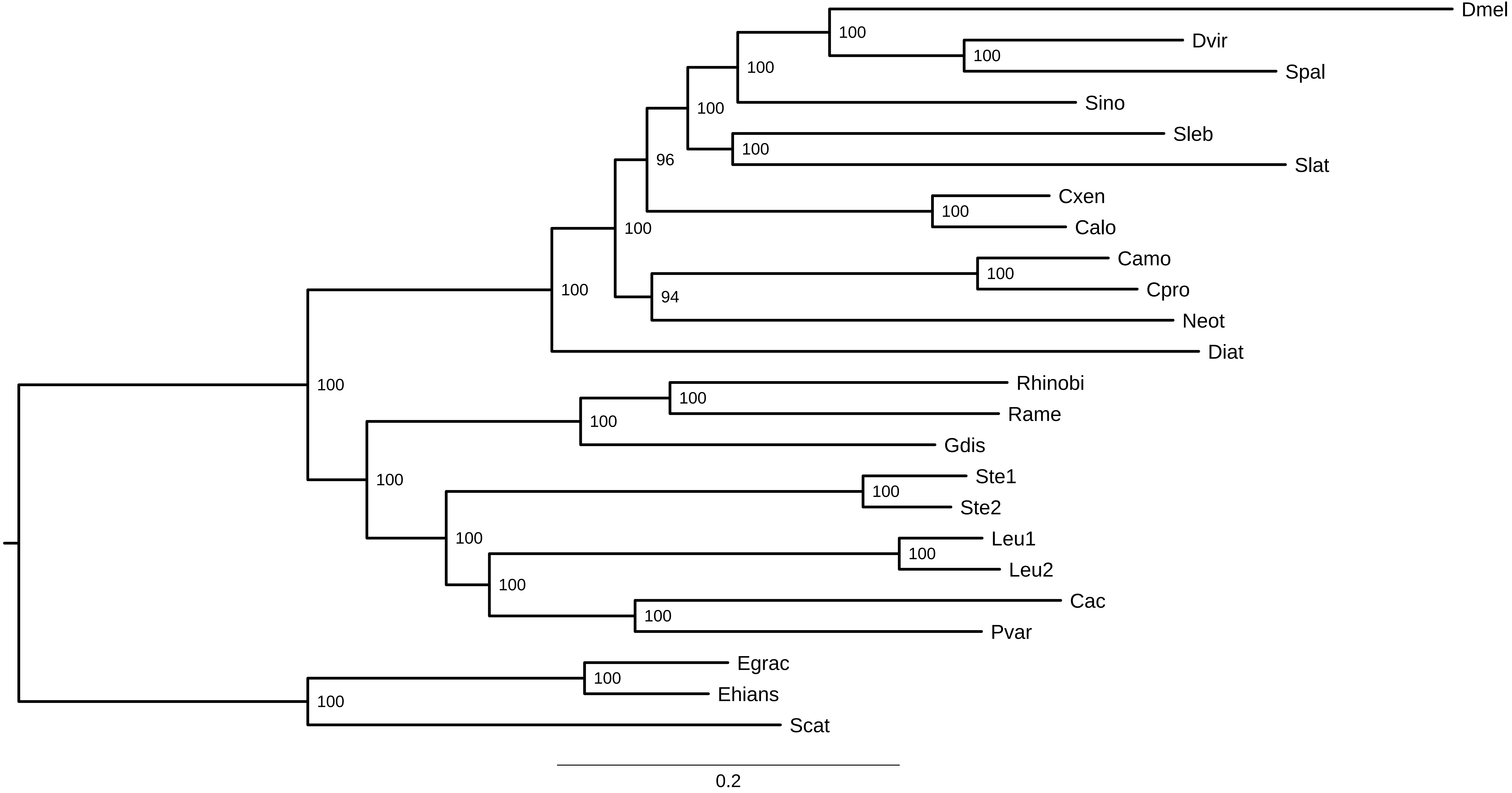
Dmel
Dvir
Spal
Sino
Sleb
Slat
Cxen
Calo
Camo
Cpro
Neot
Diat
Rhinobi
Rame
Gdis
Ste1
Ste2
Leu1
Leu2
Cac
Pvar
Egrac
Ehians
Scat
100
96
94
0.2

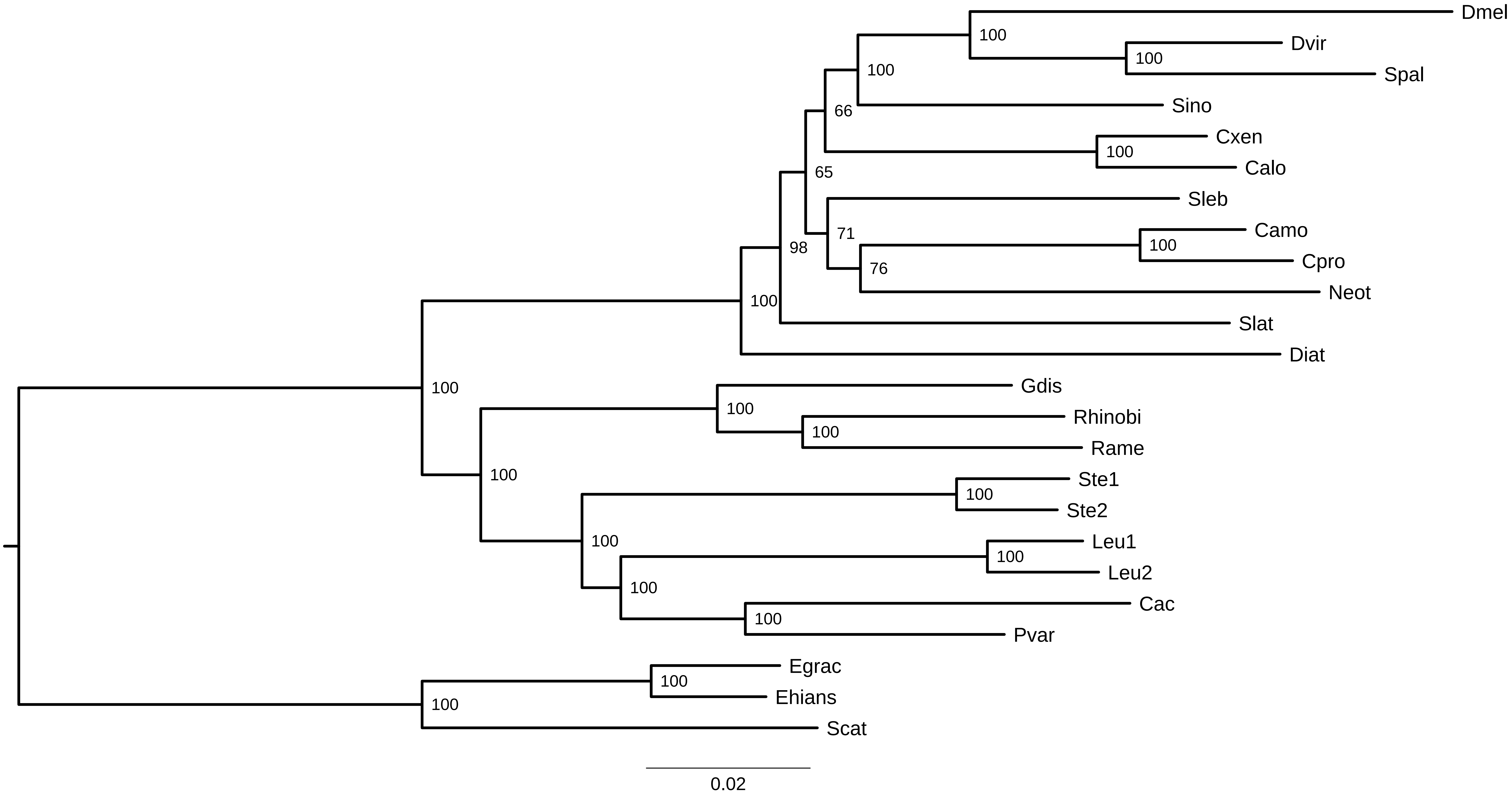
Dmel
100
Dvir
100
Spal
100
Sino
66
Cxen
100
Calo
65
Sleb
Camo
71
100
Cpro
76
98
Neot
100
Slat
Diat
100
Gdis
100
Rhinobi
100
Rame
100
Ste1
100
Ste2
Leu1
100
100
Leu2
100
Cac
100
Pvar
Egrac
100
Ehians
100
Scat
0.02

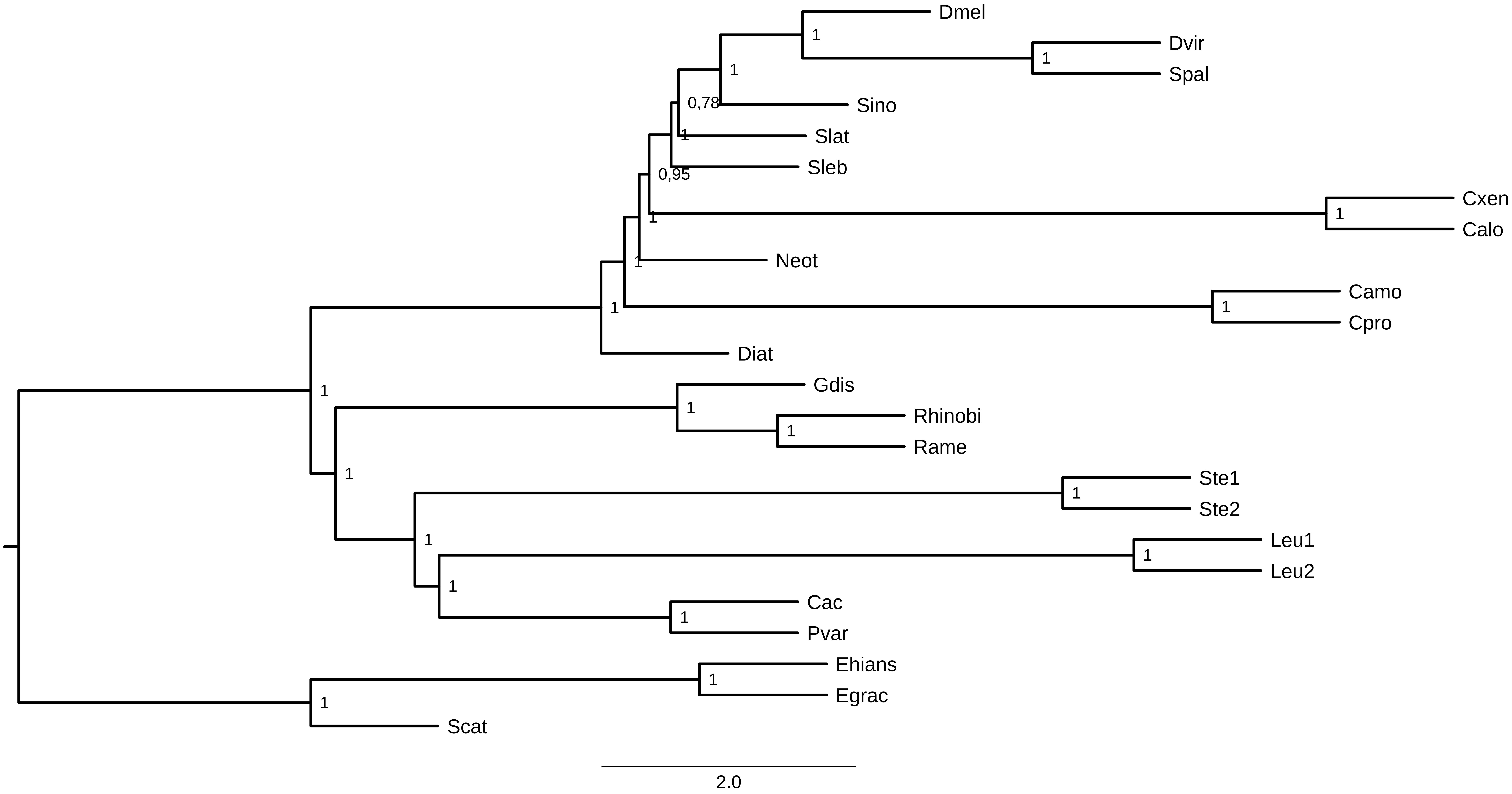

Dmel
Dvir
Spal
Sino
Slat
Sleb
Cxen
Calo
Neot
Camo
Cpro
Diat
Gdis
Rhinobi
Rame
Ste1
Ste2
Leu1
Leu2
Cac
Pvar
Ehians
Egrac
Scat
0,78
0,95
1
2.0

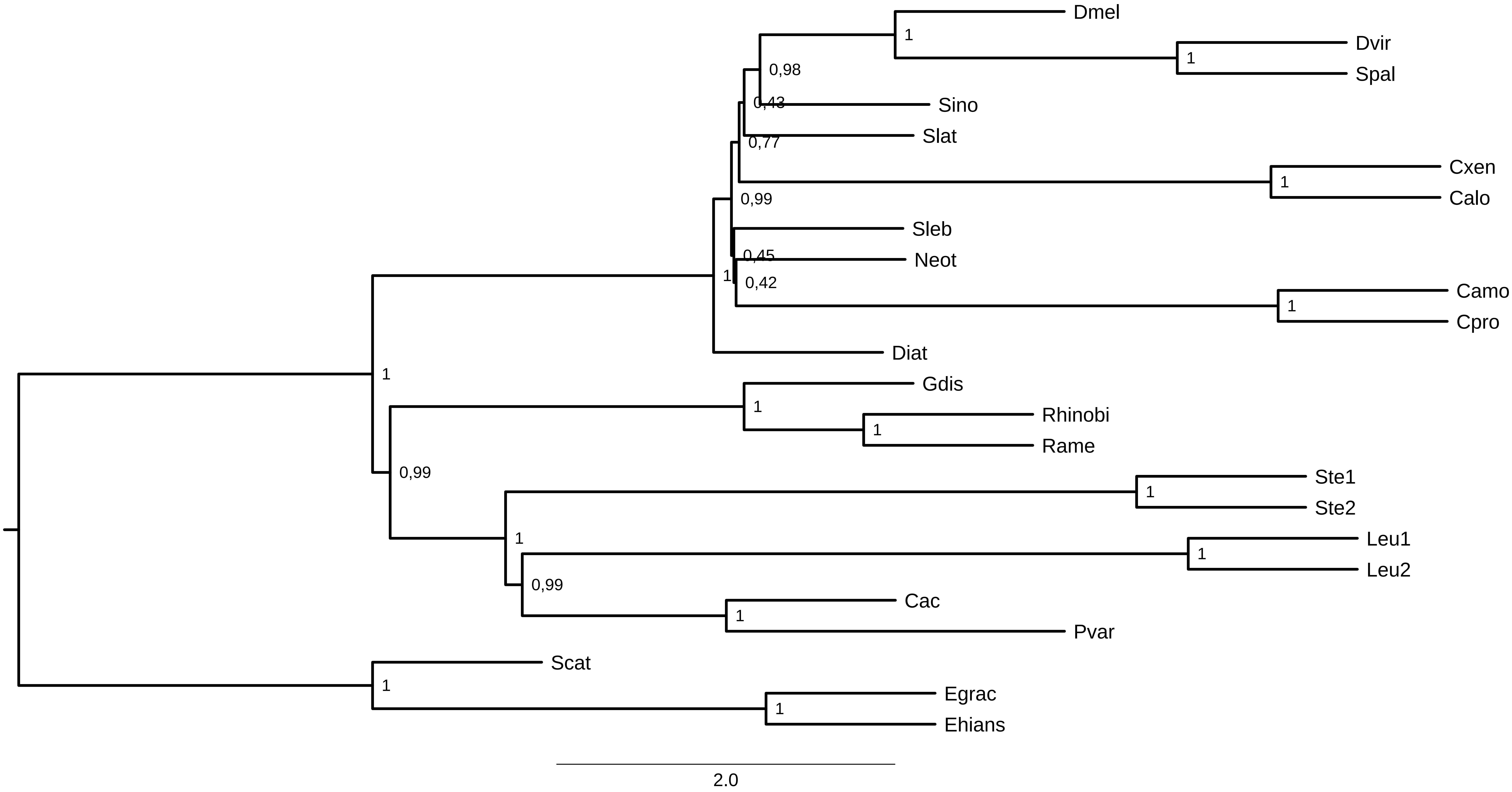
Dmel
1
Dvir
1
Spal
0,98
Sino
0,43
Slat
0,77
Cxen
1
Calo
0,99
Sleb
0,45
Neot
1
0,42
Camo
1
Cpro
Diat
1
Gdis
1
Rhinobi
1
Rame
0,99
Ste1
1
Ste2
1
Leu1
1
Leu2
0,99
Cac
1
Pvar
Scat
1
Egrac
1
Ehians
2.0

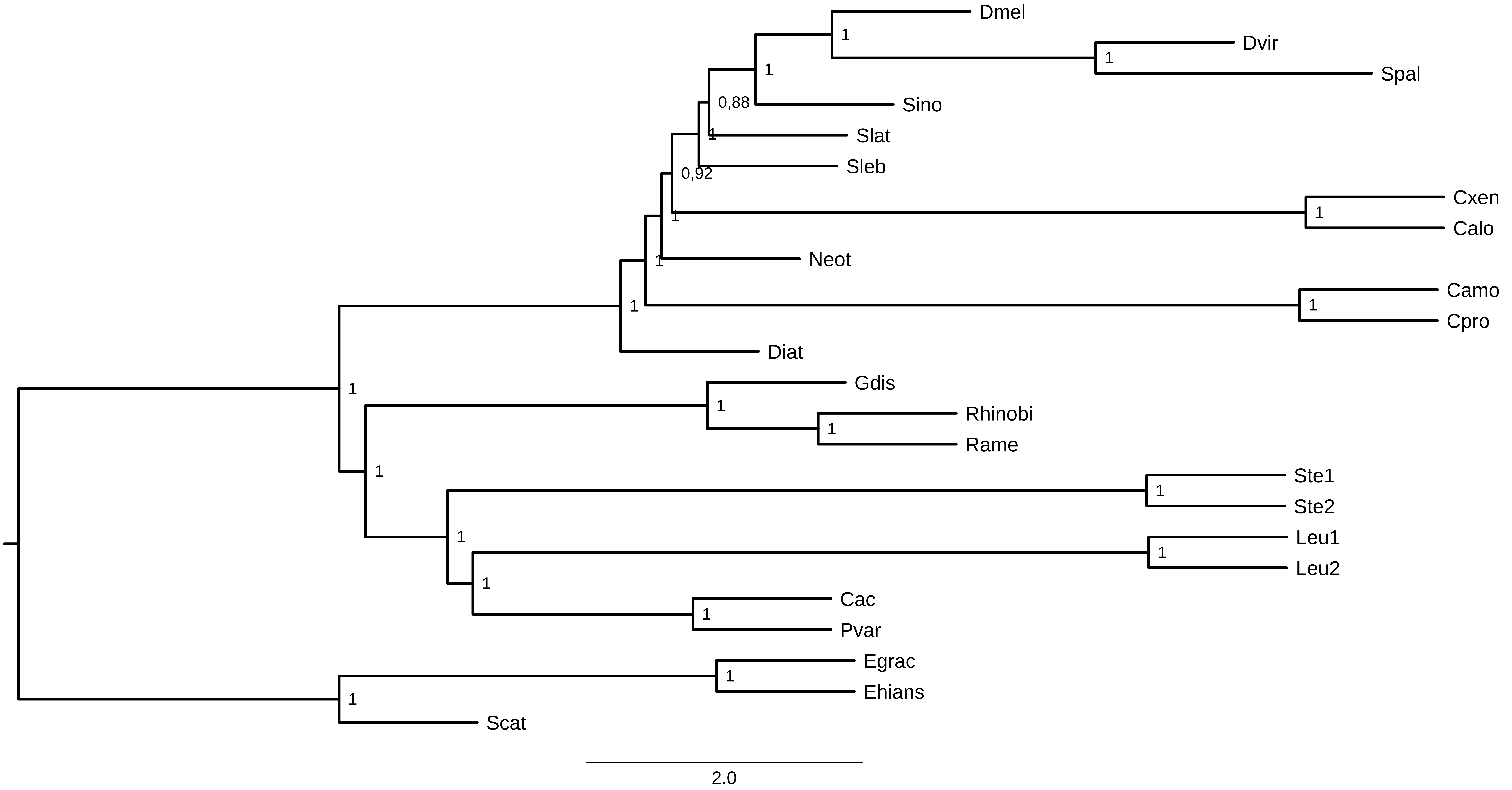
Dmel
Dvir
Spal
Sino
Slat
Sleb
Cxen
Calo
Neot
Camo
Cpro
Diat
Gdis
Rhinobi
Rame
Ste1
Ste2
Leu1
Leu2
Cac
Pvar
Egrac
Ehians
Scat
0,88
0,92
2.0

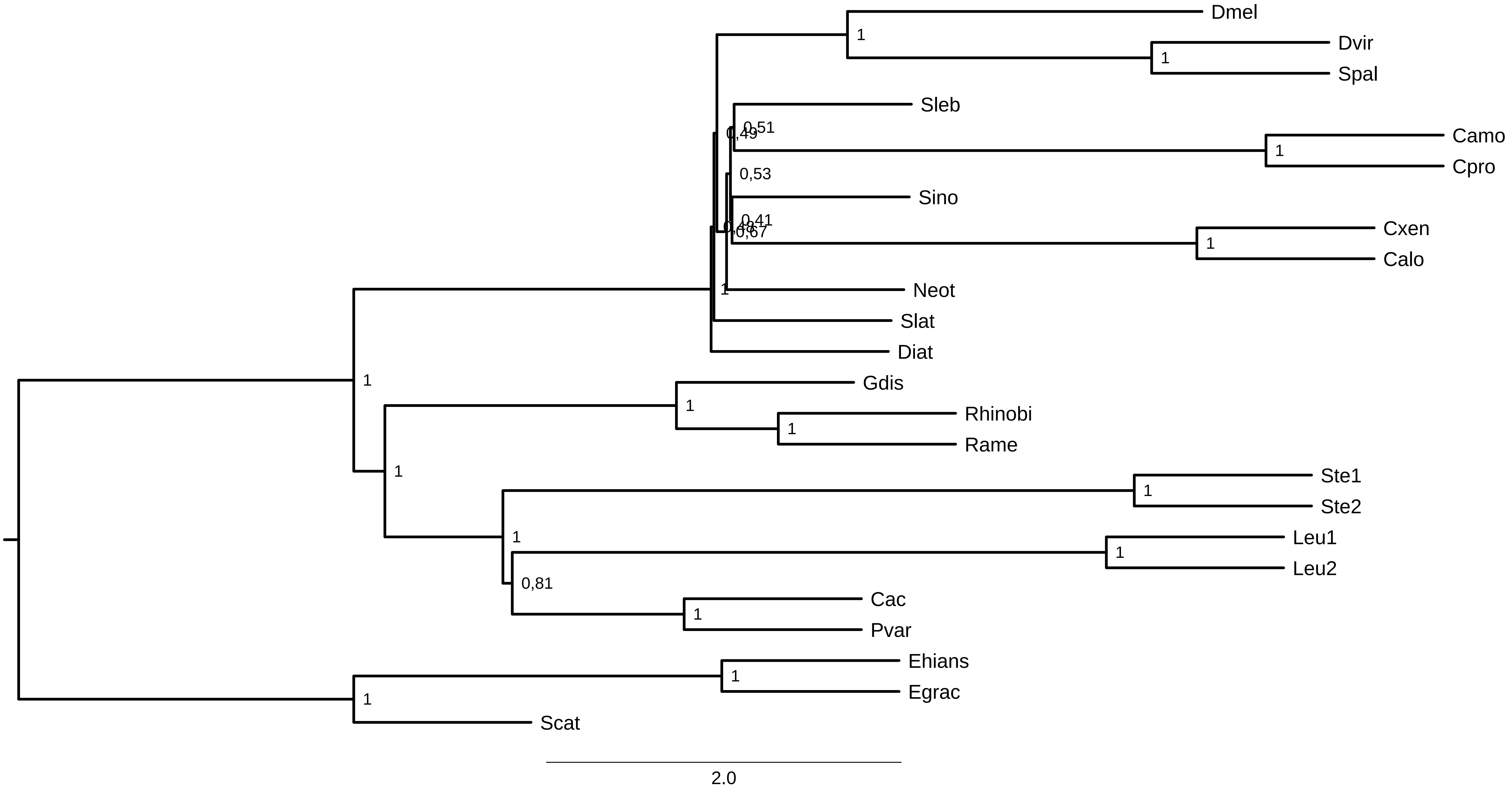

Dmel
Dvir
Spal
Sleb
Camo
Cpro
Sino
Cxen
Calo
Neot
Slat
Diat
Gdis
Rhinobi
Rame
Ste1
Ste2
Leu1
Leu2
Cac
Pvar
Ehians
Egrac
Scat
0,51
0,49
0,53
0,41
0,67
0,81
2.0

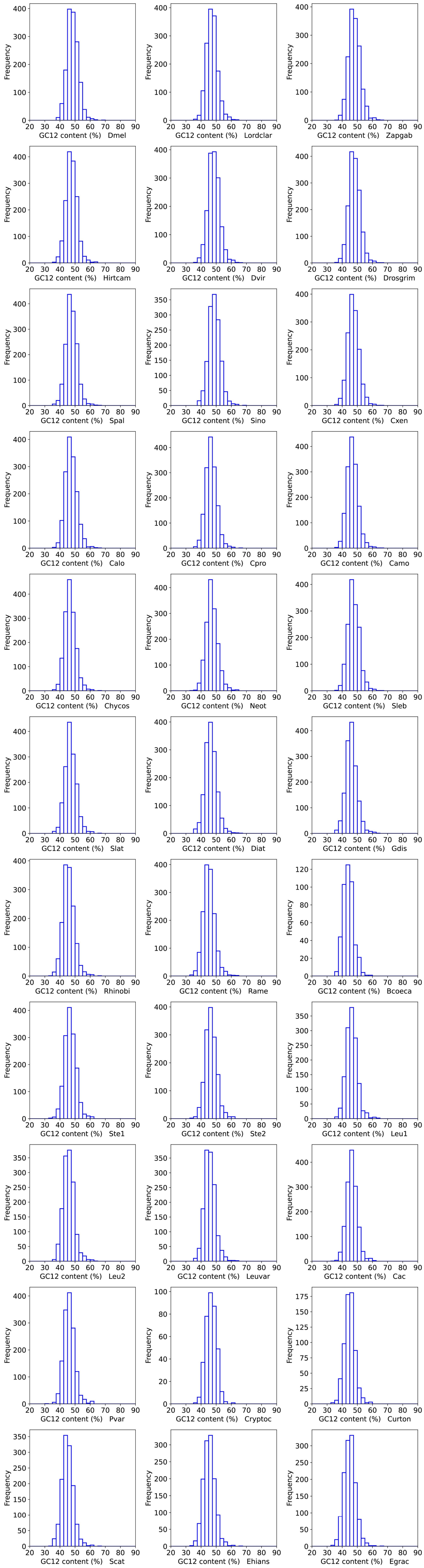
Frequency
GC12 content (%)
Dmel
Lordclar
Zapgab
Hirtcam
Dvir
Drosgrim
Spal
Sino
Cxen
Calo
Cpro
Camo
Chycos
Neot
Sleb
Slat
Diat
Gdis
Rhinobi
Rame
Bcoeca
Ste1
Ste2
Leu1
Leu2
Leuvar
Cac
Pvar
Cryptoc
Curton
Scat
Ehians
Egrac

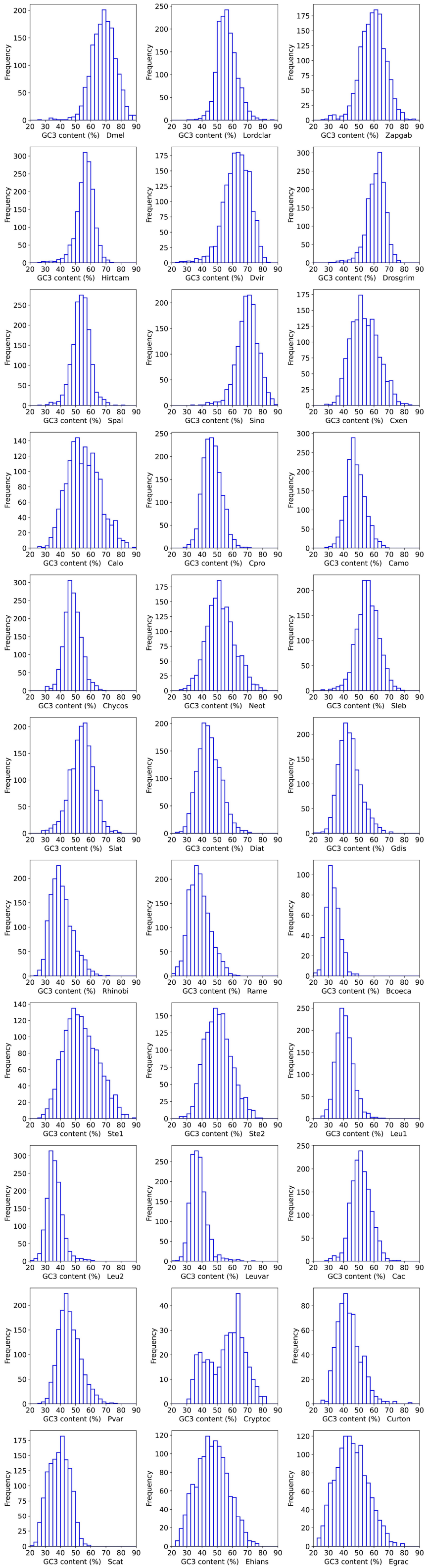
Frequency
GC3 content (%) Dmel
GC3 content (%) Lordclar
GC3 content (%) Zapgab
GC3 content (%) Hirtcam
GC3 content (%) Dvir
GC3 content (%) Drosgrim
GC3 content (%) Spal
GC3 content (%) Sino
GC3 content (%) Cxen
GC3 content (%) Calo
GC3 content (%) Cpro
GC3 content (%) Camo
GC3 content (%) Chycos
GC3 content (%) Neot
GC3 content (%) Sleb
GC3 content (%) Slat
GC3 content (%) Diat
GC3 content (%) Gdis
GC3 content (%) Rhinobi
GC3 content (%) Rame
GC3 content (%) Bcoeca
GC3 content (%) Ste1
GC3 content (%) Ste2
GC3 content (%) Leu1
GC3 content (%) Leu2
GC3 content (%) Leuvar
GC3 content (%) Cac
GC3 content (%) Pvar
GC3 content (%) Cryptoc
GC3 content (%) Curton
GC3 content (%) Scat
GC3 content (%) Ehians
GC3 content (%) Egrac

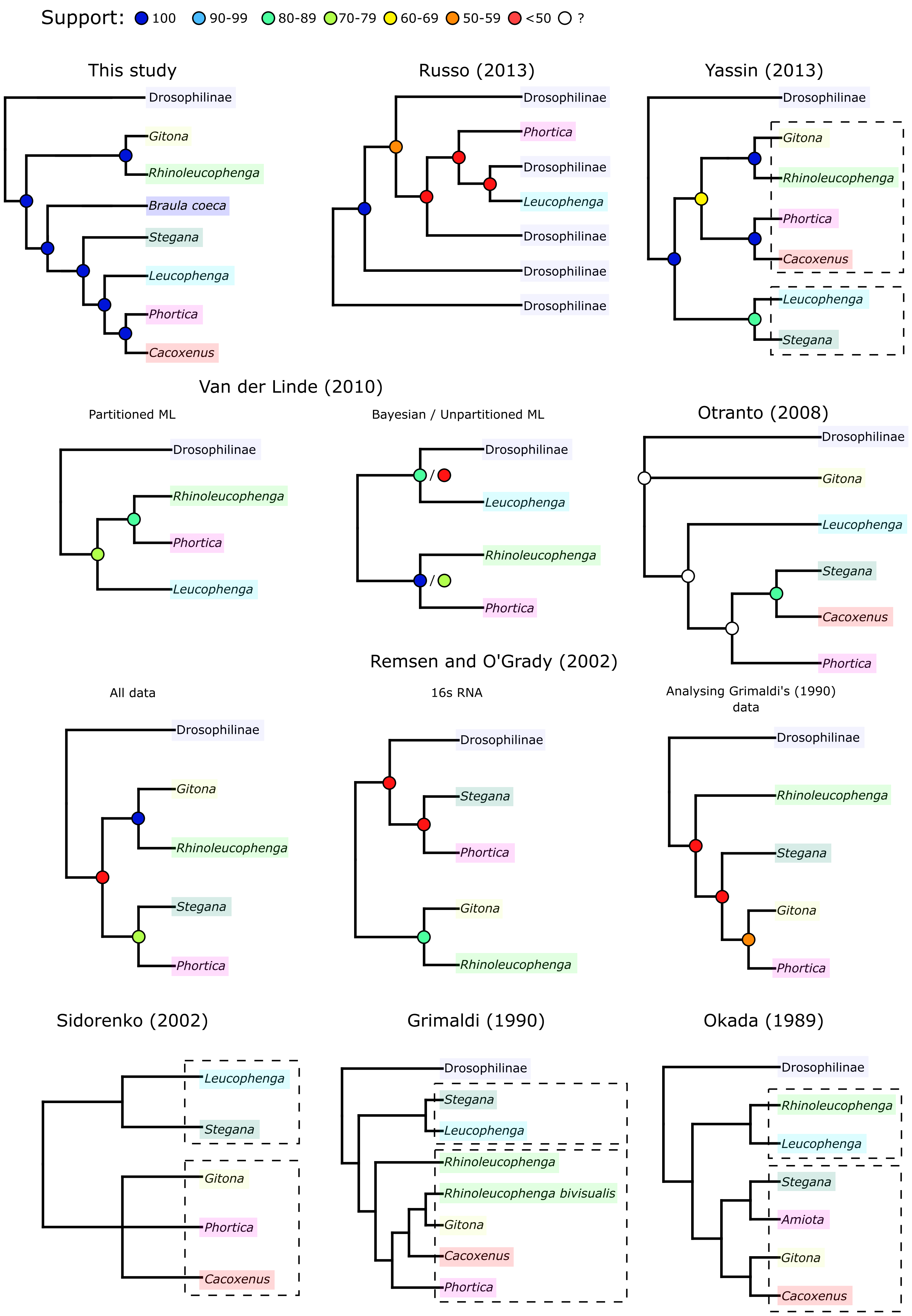

Support: 100 90-99 80-89 70-79 60-69 50-59 <50 ?
This study
Drosophilinae
Gitona
Rhinoleucophenga
Braula coeca
Stegana
Leucophenga
Phortica
Cacoxenus
Russo (2013)
Drosophilinae
Phortica
Drosophilinae
Leucophenga
Drosophilinae
Drosophilinae
Drosophilinae
Yassin (2013)
Drosophilinae
Gitona
Rhinoleucophenga
Phortica
Cacoxenus
Leucophenga
Stegana
Van der Linde (2010)
Partitioned ML
Drosophilinae
Rhinoleucophenga
Phortica
Leucophenga
Bayesian / Unpartitioned ML
Drosophilinae
Leucophenga
Rhinoleucophenga
Phortica
Otranto (2008)
Drosophilinae
Gitona
Leucophenga
Stegana
Cacoxenus
Phortica
Remsen and O'Grady (2002)
All data
Drosophilinae
Gitona
Rhinoleucophenga
Stegana
Phortica
16s RNA
Drosophilinae
Stegana
Phortica
Gitona
Rhinoleucophenga
Analysing Grimaldi's (1990) data
Drosophilinae
Rhinoleucophenga
Stegana
Gitona
Phortica
Sidorenko (2002)
Leucophenga
Stegana
Gitona
Phortica
Cacoxenus
Grimaldi (1990)
Drosophilinae
Stegana
Leucophenga
Rhinoleucophenga
Rhinoleucophenga bivisualis
Gitona
Cacoxenus
Phortica
Okada (1989)
Drosophilinae
Rhinoleucophenga
Leucophenga
Stegana
Amiota
Gitona
Cacoxenus

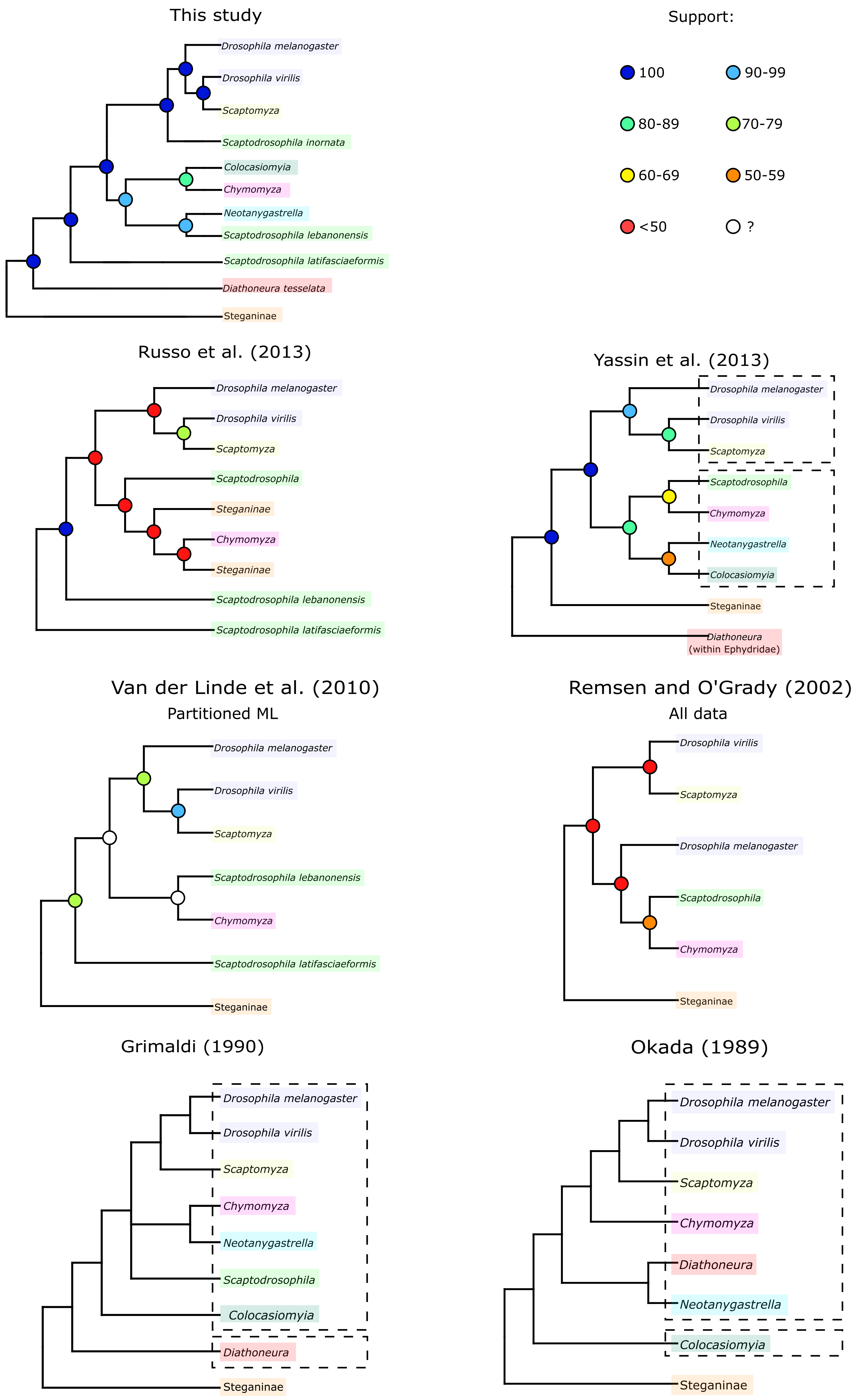
This study
Drosophila melanogaster
Drosophila virilis
Scaptomyza
Scaptodrosophila inornata
Colocasiomyia
Chymomyza
Neotanygastrella
Scaptodrosophila lebanonensis
Scaptodrosophila latifasciaeformis
Diathoneura tesselata
Steganinae
Support:
100
90-99
80-89
70-79
60-69
50-59
<50
?
Russo et al. (2013)
Drosophila melanogaster
Drosophila virilis
Scaptomyza
Scaptodrosophila
Steganinae
Chymomyza
Steganinae
Scaptodrosophila lebanonensis
Scaptodrosophila latifasciaeformis
Yassin et al. (2013)
Drosophila melanogaster
Drosophila virilis
Scaptomyza
Scaptodrosophila
Chymomyza
Neotanygastrella
Colocasiomyia
Steganinae
Diathoneura
(within Ephydridae)
Van der Linde et al. (2010)
Partitioned ML
Drosophila melanogaster
Drosophila virilis
Scaptomyza
Scaptodrosophila lebanonensis
Chymomyza
Scaptodrosophila latifasciaeformis
Steganinae
Remsen and O'Grady (2002)
All data
Drosophila virilis
Scaptomyza
Drosophila melanogaster
Scaptodrosophila
Chymomyza
Steganinae
Grimaldi (1990)
Drosophila melanogaster
Drosophila virilis
Scaptomyza
Chymomyza
Neotanygastrella
Scaptodrosophila
Colocasiomyia
Diathoneura
Steganinae
Okada (1989)
Drosophila melanogaster
Drosophila virilis
Scaptomyza
Chymomyza
Diathoneura
Neotanygastrella
Colocasiomyia
Steganinae

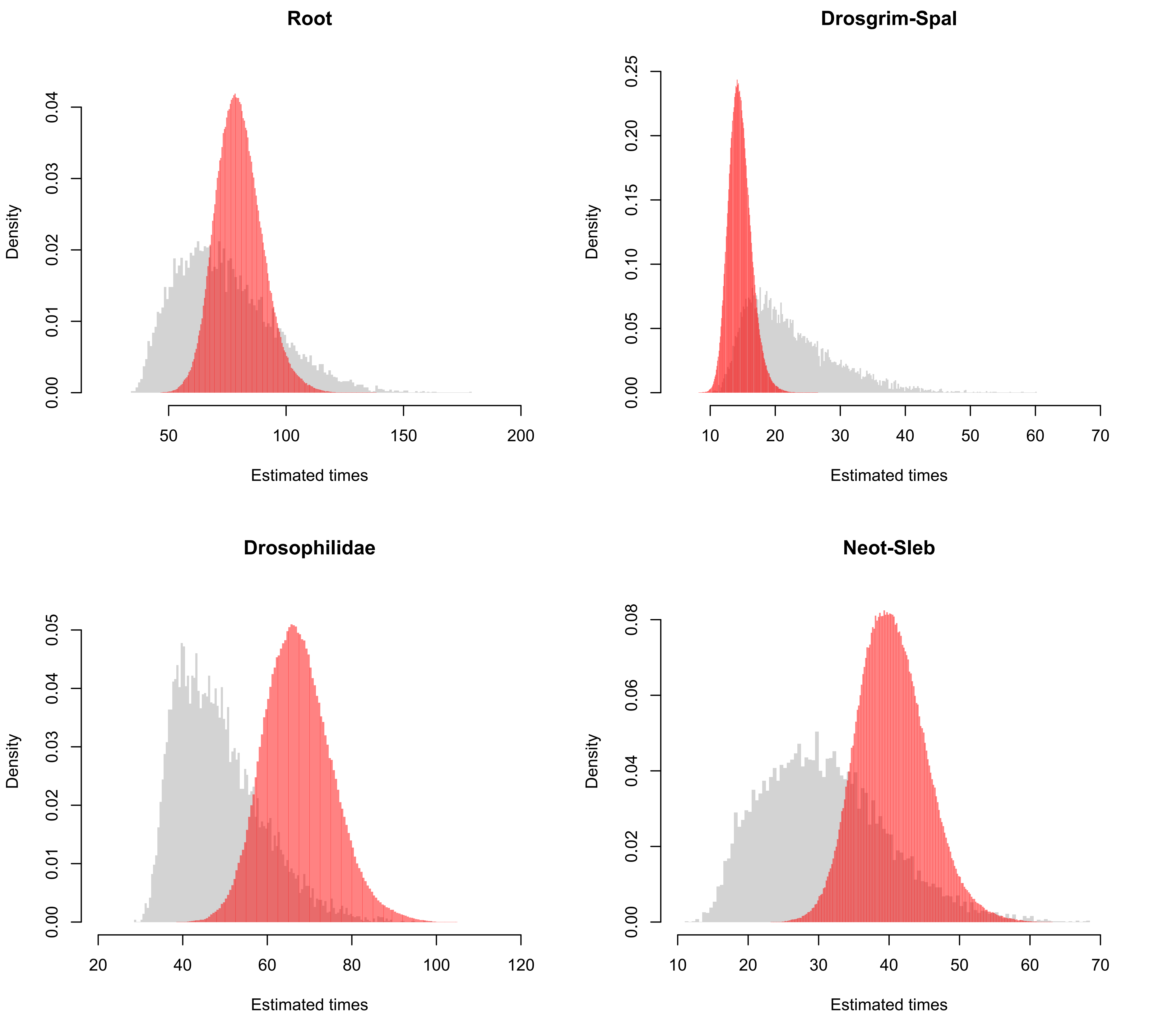
Root
Drosgrim-Spal
Drosophilidae
Neot-Sleb
Density
Estimated times
0.00
0.01
0.02
0.03
0.04
50
100
150
200
0.05
0.10
0.15
0.20
0.25
10
20
30
40
50
60
70
0.06
0.08
120

Supplementary figures

Figs. S1-S18. Species trees inferred by phylogenomic analysis using distinct datasets and methods. Table 1 summarizes the methods and dataset used in each inference (figures S1-S18 represent analyses 1-18 respectively). Species names: Dmel: *Drosophila melanogaster*; Lordclar: *Lordiphosa clarofinis*; Zapgab: *Zaprionus gabonicus*; Hirtcam: *Hirtodrosophila cameraria*; Dvir: *Drosophila virilis*; Drosgim: *Drosophila grimshawi*; Spal: *Scaptomyza palmae*; Sino: *Scaptodrosophila inornata*; Sleb: *Scaptodrosophila lebanonensis*; Slat: *Scaptodrosophila latifasciaeformis*; Cxen; *Colocasyomyia xenalocasiae*; Calo: *Colocasiomyia alocasiae*; Neot: *Neotanygastrella* sp.; Cpro: *Chymomyza procnemis*; Camo: *Chymomyza amoena*; Chycos: *Chymomyza costata*; Diat: *Diathoneura tesselata*; Gdis: *Gitona distigma*; Rhinobi: *Rhinoleucophenga cf. bivisualis*; Rame: *Rhinoleucophenga americana*; Bcoeca: *Braula coeca*; Ste1: *Stegana* sp. *1*; Ste2: *Stegana* sp. *2*; Leu1: *Leucophenga* sp. *1*; Leu2: *Leucophenga* sp. *2*; Pvar: *Phortica variegata*; Cac: *Cacoxenus indagator*; Cryptoc: *Cryptochetum* sp.; Curton: *Curtonotum* sp.; Egrac: *Ephydra gracilis*; Ehians: *Ephydra hians*; Scat: *Scatella* sp.

Fig S19. Variation in GC composition (codon positions 1 + 2) among the 33 species (comprehensive dataset). See Table S1 for the full species names.

Fig S20. Variation in GC composition (third codon position) among the 33 species (comprehensive dataset). See Table S1 for the full species names.

Fig. S21. Topological relationships of Steganinae lineages derived from different studies. Genera are highlighted with distinct colors, and only the taxa used in the present work are shown. When a genus was recovered as non-monophyletic, more than one species was represented. Node colored circles depict the support value (bootstrap or posterior

probability) for its ancestral branch in the original study. White circles represent nodes for which support was not provided in the original publication. Sidorenko (2002), Grimaldi (1990), and Okada (1989) did not provide node support values. Dashed boxes indicate the tribes proposed in the respective studies. *Amiota* is painted with the same color as *Phortica* in Okada's (1989) tree because *Phortica* was considered a subgenus of *Amiota* in that work.

Fig. S22. Topological relationships of Drosophilinae lineages derived from different studies. Genera are highlighted with distinct colors, and only the taxa used in the present work are shown. When a genus was recovered as non-monophyletic, more than one species is represented. Colored circles depict the support (bootstrap or posterior probability) for its ancestral branch in the original study. White circles represent nodes for which support was not provided in the original publication. DeSalle and Grimaldi (1991) and Okada (1989) did not provide node support values. Dashed boxes indicate the tribes proposed in the respective studies.

Fig. S23. Effective priors (gray) and posterior distributions (red) for the four fossil-calibrated nodes. In all cases, posterior densities are narrower and shifted relative to priors, indicating that divergence time estimates were primarily informed by molecular data rather than calibration bounds.
