# Supplementary material for: Timing and Pattern of Early Diversification in Drosophilidae (Diptera)
Source: Mol Biol Evol. 2025 Oct 23;42(11):msaf269. doi: 10.1093/molbev/msaf269 (PMC12603360; doi:10.1093/molbev/msaf269)
Supplement: msaf269_Supplementary_Data [file msaf269_supplementary_data.zip › MBE25-0386_Supplementary_Tables_S1-S6.pdf]

Supplementary Tables

Table S1. Variation in GC composition among the 33 species (comprehensive dataset).

Species (abridged name)	GC12 (mean ± std)	GC3 (mean ± std)	Number of genes
Drosophila melanogaster (Dmel)	48.39 ± 3.66	68.38 ± 7.96	1511
Lordiphosa clarofinis (Lordclar)	47.18 ± 3.67	56.47 ± 6.43	1450
Zaprionus gabonicus (Zapgab)	48.04 ± 3.89	59.25 ± 8.37	1510
Hirtodrosophila cameraria(Hirtcam)	47.63 ± 3.71	56.21 ± 5.97	1522
Drosophila virilis (Dvir)	48.37 ± 3.86	62.93 ± 8.60	1553
Drosophila grimshawi (Drosgrim)	48.06 ± 3.79	61.36 ± 6.12	1557
Scaptomyza palmae (Spal)	47.55 ± 3.75	53.65 ± 5.96	1529
Scaptodrosophila inornata (Sino)	48.75 ± 3.86	69.89 ± 7.20	1410
Colocasiomyia xenalocasiae (Cxen)	47.33 ± 3.79	53.84 ± 8.94	1445
Colocasiomyia alocasiae (Calo)	47.39 ± 3.86	55.16 ± 10.54	1496
Chymomyza procnemis (Cpro)	46.67 ± 3.67	46.70 ± 6.03	1512
Chymomyza amoena (Camo)	46.75 ± 3.70	47.96 ± 6.04	1511
Chymomyza costata (Chycos)	46.75 ± 3.69	48.08 ± 5.62	1545
Neotanygastrella sp. (Neot)	47.07 ± 3.91	52.37 ± 9.14	1474
Scaptodrosophila lebanonensis (Sleb)	47.47 ± 3.80	55.78 ± 7.31	1479
Scaptodrosophila latifasciaeformis (Slat)	47.09 ± 3.84	54.06 ± 7.71	1464
Diathoneura tesselata (Diat)	46.43 ± 3.91	44.71 ± 7.51	1449
Gitona distigma (Gdis)	46.05 ± 3.77	44.05 ± 7.19	1476
Rhinoleucophenga bivisualis (Rhinobi)	45.72 ± 3.83	40.67 ± 7.17	1444
Rhinoleucophenga americana (Rame)	45.25 ± 3.88	38.30 ± 6.97	1492
Braula coeca (Bcoeca)	44.01 ± 3.41	32.89 ± 4.51	445
Stegana sp.1 (Ste1)	46.80 ± 3.83	53.69 ± 10.99	1476
Stegana sp.2 (Ste2)	46.55 ± 3.84	50.53 ± 8.77	1427
Leucophenga sp.1 (Leu1)	46.28 ± 3.69	40.67 ± 5.62	1326
Leucophenga sp.2 (Leu2)	45.81 ± 3.71	36.24 ± 5.22	1392
Leucophenga varia (Leuvar)	45.78 ± 3.66	37.57 ± 5.51	1386
Cacoxenus indagator (Cac)	46.38 ± 3.56	51.39 ± 6.57	1459
Phortica variegata (Pvar)	46.15 ± 3.70	46.08 ± 7.27	1427
Cryptochetum sp. (Cryptoc)	46.63 ± 3.45	55.87 ± 11.86	371
Curtonotum sp. (Curton)	44.90 ± 3.50	43.52 ± 7.77	634
Scatella sp. (Scat)	45.09 ± 3.82	39.10 ± 6.73	1289
Ephydra hians (Ehians)	45.39 ± 3.84	46.78 ± 10.30	1259
Ephydra gracilis (Egrac)	45.06 ± 3.83	45.44 ± 10.30	1288

Table S2. Summary of whether each tribe proposed in the literature (Okada 1989; Grimaldi 1990; Sidorenko 2002; Yassin 2013) for Drosophilidae was recovered as monophyletic in our dataset. Tribes represented by a single genus were not formally tested for monophyly.

Subfamily	Tribe (Original Author)	Monophyly Recovered
**Drosophilinae**	Drosophilini (Okada, 1989)	No
	Drosophilini (Grimaldi, 1990)	No
	Drosophilini (Yassin, 2013)	Yes
	Colocasiomyini (Okada, 1989)	Not tested (1 genus)
	Colocasiomyini (Yassin, 2013)	Yes
	Cladochaetini (Grimaldi, 1990)	Not tested (1 genus)
	Microdrosophilini (Okada, 1989)	Not sampled
	Hypselothyrini (Okada, 1989)	Not sampled
	Dettopsomyini (Okada, 1989)	Not sampled
**Steganinae**	Steganini (Okada, 1989)	No
	Leucophengini (Okada, 1989)	No
	Steganini (Sidorenko, 2002)	No
	Gitonini (Sidorenko, 2002)	No
	Steganini (Grimaldi, 1990)	No
	Gitonini (Grimaldi, 1990)	No
	Steganini (Yassin, 2013)	No
	Gitonini (Yassin, 2013)	No

Table S3. Sources of the genome and transcriptome assemblies used.

Species	Strain (source)*	Sequencing	Assembly
**Genomes:**			
**Drosophilinae:**			
Drosophila melanogaster	ISO1 (BDGP - #2057)	(Adams et al., 2000)	(Adams et al. 2000; Hoskins et al. 2015)
Drosophila virilis	(NDSSC - #15010-1051.87)	(Clark et al., 2007)	(Clark et al., 2007)
Drosophila grimshawi	(Kim et al. 2021) BioSample: SAMN16729613	(Kim et al. 2021)	(Kim et al. 2021)
Hirtodrosophila cameraria	BioSample: SAMEA12110596	Wellcome Sanger Institute. Bioproject: PRJEB60601	Wellcome Sanger Institute. Bioproject: PRJEB60601
Lordiphosa clarofinis	(Kim et al. 2021) BioSample: SAMN16729635	(Kim et al. 2021)	(Kim et al. 2021)
Zaprionus gabonicus	(Kim et al. 2021) BioSample: SAMN16729655	(Kim et al. 2021)	(Kim et al. 2021)
Scaptomyza palmae	(SD-DSSC - # 33000-2681.01)**	this work	this work
Scaptodrosophila inornata	Australia – collected by Michael Polak and William T. Starmer**	this work	this work
Scaptodrosophila lebanonensis	(Vicoso and Bachtrog, 2015) BioSample: SAMN03220570	(Vicoso and Bachtrog, 2015)	this work
Scaptodrosophila latifasciaeformis	(SD-DSSC -#11030-0061.01)**	this work	this work
Colocasiomyia xenalocasiae	DSEU - #E16801 ***	(Dias et al. 2020)	this work

Colocasiomyia alocasiae	Japan – collected by T. Miyake and M. Yafuso**	this work	this work
Neotanygastrella sp.	Ubatumirim, SP, Brazil – collected by A. B. Carvalho**	this work	this work
Chymomyza amoena	SD-DSSC - #20010-2010.01 ***	(Dias et al. 2020)	this work
Chymomyza procnemis	SD-DSSC - #2000-2640.00**	this work	this work
Chymomyza costata	(Kim et al. 2021) BioSample: SAMN16729672	(Kim et al. 2021)	(Kim et al. 2021)
Diathoneura tesselata	(Collier and Armstrong 2009)**	this work	this work
**Steganinae:**			
Gitona distigma	Switzerland – collected by G. Bachli**	this work	this work
Rhinoleucophenga cf. bivisualis	Brasilia, Brazil – collected by Tidon, R.**	(Dias et al. 2020)	this work
Rhinoleucophenga americana	SD-DSSC - #70000-3000.00 **	this work	this work
Stegana sp. *1*	Itatiaia, RJ, Brazil – collected by C. Vilela and Carvalho lab members**	this work	this work
Stegana sp. *2*	Itatiaia, RJ, Brazil – collected by C. Vilela and Carvalho lab members**	this work	this work
Leucophenga sp. *1*	Ribeirão Preto, SP, Brazil – collected by Marco Gottschalk**	this work	this work
Leucophenga sp. *2*	Ribeirão Preto, SP, Brazil – collected by Marco Gottschalk**	this work	this work
Leucophenga varia	(Kim et al. 2021) BioSample: SAMN16729689	(Kim et al. 2021)	(Kim et al. 2021)
Phortica variegata	(Vicoso and Bachtrog, 2015)	(Vicoso and Bachtrog, 2015)	this work

	BioSamples: SAMN01999150 and SAMN01999151		
Cacoxenus indagator	France – collected by P. Bee	(Dias et al. 2020)	this work
**Transcriptomes:**			
**Braulidae:**			
Braula coeca	(Bayless et al. 2021) BioSample: SAMN03753801	(Bayless et al. 2021)	(Bayless et al. 2021)
**Cryptochetidae:**			
Cryptochetum sp.	(Bayless et al. 2021) BioSample: SAMN12301943	(Bayless et al. 2021)	(Bayless et al. 2021)
**Curtonotidae:**			
Curtonotum sp.	(Bayless et al. 2021) BioSample: SAMN13111546	(Bayless et al. 2021)	(Bayless et al. 2021)
**Ephydridae:**			
Scatella sp.	Niterói, RJ, Brazil - collected by Fabiana Uno**	this work	this work
Ephydra hians	(Vicoso and Bachtrog, 2015) BioSample: SAMN03220490	(Vicoso and Bachtrog, 2015)	this work
Ephydra gracilis	(Vicoso and Bachtrog, 2015) BioSamples: SAMN03220555 and SAMN03220554	(Vicoso and Bachtrog, 2015)	this work

* BDGP, Berkeley Drosophila Genome Project (USA); SD-DSSC, San Diego Drosophila Species Stock Center (USA) (currently the NDSSC, but the strain is not available on their website); NDSSC, National Drosophila Species Stock Center (USA); DSEU, *Drosophila* Stocks of Ehime University (Japan).

** Further information in the Supplementary File 2.

*** For further information, see Dias et al. (2020)

Table S4. Genome assemblies' statistics.

SPECIES	ASSEMBLER	NUMBER OF CONTIGS	TOTAL LENGTH (BP)	LARGEST CONTIG (BP)	N50	AVERAGE COVERAGE**	NUMBER OF CONTIGS (>=500 BP)	TOTAL LENGTH (>=500 BP)
**Drosophilinae:**								
Scaptomyza palmae	SPADES	11,010	121,519,870	1,505,454	251,498	106.2	5,227	119,848,127
Scaptodrosophila inornata	PLATANUS	39,054	139,997,119	265,398	40,328	99.9	11,351	139,997,119
Scaptodrosophila lebanonensis	SPADES	66,980	205,400,339	177,787	16,008	15.1	33,591	195,025,247
Scaptodrosophila latifasciaeformis	SPADES	24,642	152,883,190	583,106	65,431	69.2	13,791	149,496,486
Colocasiomyia xenalocasiae	SPADES	26,950	165,582,508	129,086	13,543	9.6	20,477	163,698,484
Colocasiomyia alocasiae	SPADES	39,460	180,792,693	232,397	25,145	16.9	18,874	174,630,484
Neotanygastrella sp.	PLATANUS	29,379	145,146,137	945,658	147,531	94.1	5,843	138,999,993
Chymomyza amoena	SPADES	80,028	339,155,042	856,182	14,621	34.6	45,663	328,869,871
Chymomyza procnemis	PLATANUS	17,290	240,213,080	3,897,372	510,272	240.9	2,471	236,364,858
Diathoneura tesselata	SPADES	23,983	159,687,459	413,958	25,989	46.8	17,002	157,559,494
**Steganinae:**								
Gitona distigma	SPADES	35,167	185,220,612	246,328	35,161	30.5	16,051	179,230,851
Rhinoleucophenga cf. bivisualis	SPADES	232,444	309,167,701	236,166	6,622	17.4	109,742	274,873,270
Rhinoleucophenga americana	SPADES	17,293	169,782,492	810,087	112,908	38	8,064	167,065,303
Stegana sp. *1*	PLATANUS	19,590	171,248,776	1,131,285	125,160	107.9	3,942	167,367,154
Stegana sp. *2*	SPADES	151,931	222,727,573	203,985	9,403	45.3	70,587	195,690,700
Leucophenga sp. *1*	SPADES	198,892	418,105,974	374,876	5,612	52.2	128,802	396,628,313
Leucophenga sp. *2*	SPADES	291,893	474,127,266	160,052	3,425	24.2	188,458	442,277,282
Phortica variegata	SPADES	25,994	141,466,599	86,319	12,965	4.4	22,040	140,207,858
Cacoxenus indagator	SPADES	64,687	235,986,901	323,754	38,986	71.1	25,299	224,160,150
**Ephydridae:**								
Scatella sp.	SPADES	133,104	329,097,010	640,857	8,820	54.2	89,721	314,584,807
Ephydra hians	SPADES	170,514	381,490,817	71,457	5,492	4	107,867	365,647,320
Ephydra gracilis	SPADES	106,737	390,603,768	153,101	8,469	4	74,115	381,402,812

** Calculated from the average coverage of the 10 larger contigs, as reported by SPAdes.

Table S5. BUSCO results.

	Complete BUSCOs (C)		Complete and single-copy BUSCOs (S)		Complete and duplicated BUSCOs (D)		Fragmented BUSCOs (F)		Missing BUSCOs (M)	
**Species**	**n**	**%**	**n**	**%**	**n**	**%**	**n**	**%**	**n**	**%**
**Drosophilinae:**										
Drosophila melanogaster	2762	98.7%	2748	98.2%	14	0.5%	21	0.8%	16	0.5%
Drosophila virilis	2728	97.5%	2718	97.1%	10	0.4%	36	1.3%	35	1.2%
Drosophila grimshawi	2765	98.8%	2751	98.3%	14	0.5%	19	0.7%	15	0.5%
Hirtodrosophila cameraria	2738	97.8%	2702	96.5%	36	1.3%	28	1.0%	33	1.2%
Lordiphosa clarofinis	2707	96.7%	2633	94.1%	74	2.6%	61	2.2%	31	1.1%
Zaprionus gabonicus	2738	97.9%	2717	97.1%	21	0.8%	39	1.4%	22	0.7%
Scaptomyza palmae	2741	98.0%	2728	97.5%	13	0.5%	36	1.3%	22	0.7%
Scaptodrosophila inornata	2519	90.0%	2500	89.3%	19	0.7%	135	4.8%	145	5.2%
Scaptodrosophila lebanonensis	2613	93.4%	2594	92.7%	19	0.7%	132	4.7%	54	1.9%
Scaptodrosophila latifasciaeformis	2654	94.8%	2639	94.3%	15	0.5%	104	3.7%	41	1.5%
Colocasiomyia xenalocasiae	2504	89.5%	2494	89.1%	10	0.4%	98	3.5%	197	7.0%
Colocasiomyia alocasiae	2652	94.7%	2607	93.1%	45	1.6%	89	3.2%	58	2.1%
Neotanygastrella sp.	2669	95.4%	2661	95.1%	8	0.3%	65	2.3%	65	2.3%
Chymomyza amoena	2659	95.0%	2635	94.1%	24	0.9%	98	3.5%	42	1.5%
Chymomyza procnemis	2729	97.5%	2701	96.5%	28	1.0%	45	1.6%	25	0.9%
Chymomyza costata	2734	97.7%	2707	96.7%	27	1.0%	46	1.6%	19	0.7%
Diathoneura tessellata	2637	94.3%	2613	93.4%	24	0.9%	108	3.9%	54	1.8%
**Steganinae:**										
Gitona distigma	2685	96.0%	2661	95.1%	24	0.9%	74	2.6%	40	1.4%
Rhinoleucophenga cf. bivisualis	2594	92.7%	2557	91.4%	37	1.3%	148	5.3%	57	2.0%
Rhinoleucophenga americana	2712	96.9%	2692	96.2%	20	0.7%	62	2.2%	25	0.9%
Stegana sp. *1*	2676	95.6%	2658	95.0%	18	0.6%	71	2.5%	52	1.9%
Stegana sp.*2*	2555	91.3%	2530	90.4%	25	0.9%	167	6.0%	77	2.7%
Leucophenga sp.*1*	2375	84.9%	2348	83.9%	27	1.0%	294	10.5%	130	4.6%
Leucophenga sp.*2*	2495	89.1%	2469	88.2%	26	0.9%	224	8.0%	80	2.9%
Leucophenga varia	2615	93.4%	2527	90.3%	88	3.1%	125	4.5%	59	2.1%
Phortica variegata	2547	91.0%	2534	90.5%	13	0.5%	128	4.6%	124	4.4%
Cacoxenus indagator	2644	94.5%	2620	93.6%	24	0.9%	92	3.3%	63	2.2%

**Ephydridae:**										
Ephydra gracilis	2317	82.8%	2308	82.5%	9	0.3%	314	11.2%	168	6.0%
Ephydra hians	2244	80.2%	2230	79.7%	14	0.5%	328	11.7%	227	8.1%
Scatella sp.	2371	84.7%	2346	83.8%	25	0.9%	274	9.8%	154	5.5%
**Braulidae**										
Braula coeca	1607	57.4%	814	29.1%	793	28.3%	617	22.0%	575	20.6%
**Cryptochetidae**										
Cryptochetum sp.	2238	80.0%	551	19.7%	1687	60.3%	169	6.0%	392	14.0%
**Curtonotidae**										
Curtonotum sp.	1675	59.9%	1094	39.1%	581	20.8%	461	16.5%	663	23.6%

Table S6. Fossil information. Fossils used directly for calibration are highlighted in bold.

Drosophilidae					
**Fossil**	**Reference**	**Origin**	**Min. age (Ma)**	**Max. age (Ma)**	**Type of fossil**
**Drosophilinae:**					
Drosophila berryi	Cockerel 1923	Valle de Jesus, Colombia	0,001	0	Copal
Drosophila (Drosophila?) poinari	Grimaldi 1987	Cotui, Dominican Republic	12	0	Copal
Chymomyza primaeva	Grimaldi 1987	Dominican Republic	13,65	20,43	Amber
Hirtodrosophila paleothoracis	Grimaldi 1987	Dominican Republic	13,65	20,43	Amber
Drosophila succini (incertae sedis)	Grimaldi 1987	Dominican Republic	13,65	20,43	Amber
Miomyia io	Grimaldi 1987	Dominican Republic	13,65	20,43	Amber
Protochymomyza miocena	Grimaldi 1987	Dominican Republic	13,65	20,43	Amber
Scaptomyza dominicana	Grimaldi 1987	Dominican Republic	13,65	20,43	Amber
Drosophilinae A *incertae sedis*	Grimaldi 1987	Dominican Republic	13,65	20,43	Amber
Drosophilinae B *incertae sedis*	Grimaldi 1987	Dominican Republic	13,65	20,43	Amber
Neotanygastrella wheeleri	Grimaldi 1987	Chiapas, Mexico	15,97	23,03	Amber
Drosophila statzi	Statz 1940	Rott Formation, Germany	24	24	Amber
Drosophila blanda	Statz 1940	Rott Formation, Germany	24	24	Amber
**Steganinae:**					
Hyalistata vitrea	Grimaldi 1993	Dominican Republic	13.65	20.43	Amber
Electrophortica succini	Hennig 1965	Baltic (Denmark)	33.9	37.2	Amber

Non-Drosophilidae Cyclorrhapha					
**Fossil**	**Reference**	**Origin**	**Min. age (Ma)**	**Max. age (Ma)**	**Type of fossil**
**Schizophora:**					
**Superfamily Ephydroidea. as old or older than *Electrophortica succini***					
Protocamilla groehni	Fossilworks (Alroy 2020)	Baltic (Russia Federation)	33.9	37.2	Amber
Protocamilla succini	Fossilworks (Alroy 2020)	Baltic (Denmark and Russia Federation)	33.9	37.2	Amber
Pareuthychaeta electrica	Fossilworks (Alroy 2020)	Baltic (Denmark and Russia Federation)	33.9	37.2	Amber
Pareuthychaeta mcalpinei	Fossilworks (Alroy 2020)	Baltic (Russia Federation)	33.9	37.2	Amber
Phanerochaetum tuxeni	Fossilworks (Alroy 2020)	Baltic (Denmark and Russia Federation)	33.9	37.2	Amber
Pareuthychaeta minuta	Fossilworks (Alroy 2020)	Baltic (Russia Federation)	33.9	37.2	Amber
Pareuthychaeta eoindica	Fossilworks (Alroy 2020)	India	48.6	55.8	Amber
**Non-Ephydroidea Schizophora:**					
Eolausanites ellipticus	Fossilworks (Alroy 2020)	China	48.6	55.8	Amber
Cuterebra ascarides	Fossilworks (Alroy 2020)	United States	46.2	50.3	Compression
Heteromyza detecta	Fossilworks (Alroy 2020)	United States	46.2	50.3	Compression
Dermatobia hydropica	Fossilworks (Alroy 2020)	United States	46.2	50.3	Compression
Cuterebra bibosa	Fossilworks (Alroy 2020)	United States	46.2	50.3	Compression
Musca sp.	Fossilworks (Alroy 2020)	United States	46.2	50.3	Compression
Sciomyza disjecta	Fossilworks (Alroy 2020)	United States	46.2	50.3	Compression
Sciomyza manca	Fossilworks (Alroy 2020)	United States	46.2	50.3	Compression
Lithexorista scudderi	Fossilworks (Alroy 2020)	United States	46.2	50.3	Compression
Phytomyzites schaarschmidti	Winkler et al. 2010	Germany	47.8	47.8	Ichnofossil
Phytomyzites biliapchaensis	Winkler et al. 2010	United States	64	64.7	Ichnofossil
**Non-Schizophora:**					
**Superfamily Phoroidea**					
Opetiala shatalkini	Fossilworks (Alroy 2020)	United Kingdom	140.2	145.5	Compression
